# Toward Mechanochromic Soft Material‐Based Visual Feedback for Electronics‐Free Surgical Effectors

**DOI:** 10.1002/advs.202100418

**Published:** 2021-06-02

**Authors:** Goffredo Giordano, Mariacristina Gagliardi, Yu Huan, Marco Carlotti, Andrea Mariani, Arianna Menciassi, Edoardo Sinibaldi, Barbara Mazzolai

**Affiliations:** ^1^ Center for Micro‐BioRobotics Italian Institute of Technology Viale Rinaldo Piaggio 34 Pontedera (PI) 56025 Italy; ^2^ The BioRobotics Institute Scuola Superiore Sant'Anna Viale Rinaldo Piaggio 34 Pontedera (PI) 56025 Italy; ^3^ Department of Excellence in Robotics and AI Scuola Superiore Sant'Anna Piazza Martiri della Libertà 33 Pisa (PI) 56127 Italy; ^4^ NEST Scuola Normale Superiore and Istituto Nanoscienze Consiglio Nazionale delle Ricerche Piazza S. Silvestro, 12 Pisa (PI) 56127 Italy

**Keywords:** biomedical, mechanochromic grasper, mechanochromism, minimally invasive surgery, spiropyran, surgical grasper, visual feedback

## Abstract

A chromogenically reversible, mechanochromic pressure sensor is integrated into a mininvasive surgical grasper compatible with the da Vinci robotic surgical system. The sensorized effector, also featuring two soft‐material jaws, encompasses a mechanochromic polymeric inset doped with functionalized spiropyran (SP) molecule, designed to activate mechanochromism at a chosen pressure and providing a reversible color change. Considering such tools are systematically in the visual field of the operator during surgery, color change of the mechanochromic effector can help avoid tissue damage. No electronics is required to control the devised visual feedback. SP‐doping of polydimethylsiloxane (2.5:1 prepolymer/curing agent weight ratio) permits to modulate the mechanochromic activation pressure, with lower values around 1.17 MPa for a 2% wt. SP concentration, leading to a shorter chromogenic recovery time of 150 s at room temperature (25 °C) under green light illumination. Nearly three‐times shorter recovery time is observed at body temperature (37 °C). To the best of knowledge, this study provides the first demonstration of mechanochromic materials in surgery, in particular to sensorize unpowered surgical effectors, by avoiding dramatic increases in tool complexity due to additional electronics, thus fostering their application. The proposed sensing strategy can be extended to further tools and scopes.

## Graphical abstract

A sensorized electronics‐free surgical grasper is obtained by combining the mechanical intelligence of a soft‐jaw grasper with the material intelligence of a chromogenically reversible, mechanochromic biocompatible inset, to provide the surgical operator with visual cues for safe tissue handling. After characterizing the mechanochromic behavior, the soft mechanochromic grasper is implemented on a surgical robotic platform (da Vinci Research kit).

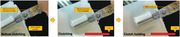

## 1 Introduction

Despite the great progresses of minimally invasive surgery (MIS) during the last decades, safe and effective tissue handling still poses formidable challenges to surgeons, who still lack even qualitative visual‐tactile indicators during tissue handling.^[^ ^1^ ^]^ Minimally invasive robotic surgery (MIRS) augment surgeons’ dexterity and accuracy, e.g., by allowing for more degrees of freedom at the end‐effector and by compensating for fatigue/tremor. However, the lack of awareness about the force that is applied in each movement still remains a crucial issue.^[^ ^2^, ^3^ ^]^ The perception of fine anatomical details is based on multiple physical characteristics, such as distributed pressure, mechanical force, and vibration.^[^ ^4^ ^]^ Therefore, researchers are endeavoring to develop novel strategies^[^ ^5^ ^]^ also based on visual/audible signals to return force cues to the operator, e.g., for grasping forces or for determining the pose of slender surgical manipulators/effectors.^[^ ^6^, ^7^ ^]^ Stimuli‐responsive components can advance MIS/MIRS, by fostering the development of a variety of tools, ranging, e.g., from graspers,^[^ ^8^ ^]^ to endoscopes,^[^ ^9^ ^]^ up to implantable and skin‐mounted devices,^[^ ^10^ ^]^ able to perform safe surgery on delicate anatomical structures.^[^ ^11^, ^12^ ^]^

At present, the most used and cutting‐edge MIRS platform worldwide is the da Vinci surgical system (Intuitive Surgical Inc., CA, USA). It is a teleoperated robot where the surgeon handles a couple of master manipulators to control robotic slave arms at the patient side, also used for intraoperative endoscopic imaging (i.e., to provide the surgeon with 3D images from inside the patient body). Additional arms mount exchangeable surgical tools.^[^ ^13^ ^]^ To augment the safety and the functionality of conventional laparoscopic tools, there is a need for efficient interfaces, sensory feedback strategies, adaptable design, and novel computational approaches.^[^ ^14^, ^15^, ^16^ ^]^ Surgical graspers provide a remarkable example: their rigid jaws, because of the miniature scale imposed by laparoscopic insertion ports (usually 5–15 mm in diameter), induce high stress concentration on the grasped tissue closer to their edges,^[^ ^17^, ^18^ ^]^ with high potential for tissue damage.^[^ ^19^ ^]^ In recent years, therefore, adaptable grasper geometries, either based on compliant jaws activated prior to clutching,^[^ ^20^ ^]^ or passively deforming during clutching,^[^ ^21^, ^22^, ^23^ ^]^ were proposed, also by the authors, aiming at respectfully interact with tissue. Furthermore, complementary investigations were carried out on sensor‐based solutions and/or computational approaches based on force reflection,^[^ ^24^, ^25^, ^26^ ^]^ aiming at improved real‐time closed‐loop haptic feedback platforms. Currently, designers implement sensing modules either outside the abdomen wall, specifically close to the actuator driving the grasper (indirect force sensing, IFS),^[^ ^27^, ^28^ ^]^ or close to the end‐effector tip (direct force sensing, DFS).^[^ ^29^, ^30^ ^]^ Theoretically, the DFS solution could improve the measurement accuracy compared to IFS, e.g., by eliminating friction/transmission effects.^[^ ^31^ ^]^ Yet DFS implementation, e.g., based on micromechatronic components (e.g., force–torque,^[^ ^32^ ^]^ capacitive,^[^ ^33^ ^]^ piezoelectric,^[^ ^34^ ^]^ and/or optoelectronic sensors^[^ ^35^ ^]^) or soft‐stretchable materials,^[^ ^36^ ^]^ lead to an increased complexity. Moreover, they must possibly comply with sterilization procedures (temperature around 120–135 °C, pressure around 200 kPa), as well as biocompatibility requirements to safely work in the human body. Conversely, IFS solutions are free from these restrictions.

In this framework, we leveraged a soft grasper devised to safely and effectively interact with tissue,^[^ ^22^, ^23^ ^]^ with the aim to augment its “mechanical intelligence” with the “material intelligence” of a novel sensing element exploiting mechanochromism.^[^ ^37^, ^38^, ^39^ ^]^ We aimed to introduce a DFS strategy based on visual feedback, without introducing additional electronics, namely to provide the operator (systematically observing the surgical scenario through the endoscopic view) with force cues based on mechanochromic‐driven color changes close to the end effector. More specifically, we integrated into the aforementioned soft grasper a mechanochromic polymeric component, i.e., a biocompatible elastomeric matrix covalently modified with a functionalized spiropyran (SP) species acting as mechanochromic unit. This force‐responsive SP molecule can reversibly switch to the planar blue/violet merocyanine (MC) form upon mechanical,^[^ ^40^, ^41^, ^42^, ^43^, ^44^ ^]^ optical, or thermal stimulation.^[^ ^36^, ^45^, ^46^, ^47^, ^48^ ^]^ The sudden changing coloration (visible absorbance variation) of the proposed DFS mechanochromic equipment, hereafter labeled as DFS‐MCE (or MCE, for simplicity), results in a time‐reversible compact optical switch able to convey information on the clutching force exerted by the grasper jaws on a grasped sample. Previous studies^[^ ^45^, ^49^, ^50^, ^51^ ^]^ report chromogenic recovery times in a mechanochromic elastomeric‐doped matrix under optical pumping down to about 10 s.^[^ ^48^ ^]^ However, such a short time was obtained for doping concentrations (0.5–0.7% wt. in a polydimethylsiloxane (PDMS) prepolymer/curing agent weight ratio 10:1) resulting in a mechanochromic activation pressure (see below) around 5 MPa, thus relatively far from the surgical scope, and specifically from biological tissue grasping. On the other hand, it was shown that an activated (i.e., mechanically compressed) mechanochromic polymeric‐doped matrix (polymethylacrylate, PMA) can retain its color for several minutes (5 min),^[^ ^52^ ^]^ thus for potential use for surgical retraction procedures requiring stable tissue holding over time.^[^ ^53^ ^]^ As a matter of fact, unless aiming to highly dynamic object manipulation for which electronic‐based sensing systems featuring a respond time in the ms range may be required,^[^ ^54^, ^55^ ^]^ the use of “material intelligence” may help avoid the complexity of miniaturized mechatronic embodiments, which, e.g., generally require wiring connections (altering mechanical compatibility), algorithmic compensation for cross‐talk loads, and biocompatible glue to support sterilization (because thermal effect causes material expansion, gage factor coefficient variation, etc.).^[^ ^56^ ^]^ Thus, the aforementioned tissue grasping/holding provides relevant surgical tasks motivating the development of electronics‐free DFS‐MCE.

Therefore, here we report a DFS‐MCE, chromogenically reversible and fully integrated in a da Vinci Research Kit (dVRK) platform,^[^ ^57^ ^]^ and able to easily alarm the surgeon about excessive interaction forces in MIRS. The color‐changing DFS‐MCE was integrated as an add‐on at the proximal end of a soft jaw grasper, in turn, integrated into the dVRK surgical robotics platform.^[^ ^22^ ^]^ The proposed study illustrates a mechanochromism‐based visual‐feedback strategy suitable for sensorizing miniaturized surgical graspers, thus taking a further step toward the development of advanced MIS/MIRS tools endowed with material intelligence.

## 2 Results and Discussions

**Figure 1:**
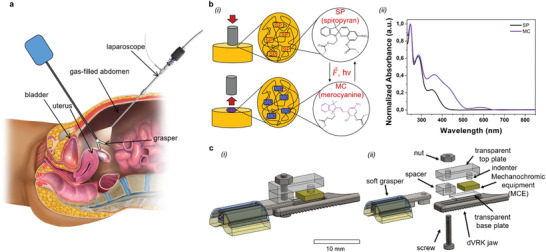
a) A pictorial representation of the anatomical districts where grasping/retraction surgical tasks are commonly performed. The blue box represents tool activation: for a surgical robotic platform like the dVRK used in this study, that box is a placeholder for teleoperated robotic slaves. The inspection endoscope is systematically used during surgery, so that 3D colored visual feedback can be leveraged to assist the surgeon. The anatomical representation is adapted by Blausen.com staff (2014). Medical gallery of Blausen Medical 2014. WikiJournal of Medicine **1** (2). DOI: 10.15347/wjm. b) (i) The mechanophore SP covalently bonded to the polymeric matrix. The mechanical indentation returns the spiro C─O bonding scission, reporting the classical SP‐to‐MC colored reversible reaction. (ii) Absorbance spectra of the SP form dissolved in dichloromethane (black line), MC (violet line). The MC spectrum reveals an absorbance peak at 560 nm, suggesting that green light can foster the reversible backward MC‐to‐SP reaction. In general, MC under white light it is favored to return to the SP form. c) (i) Assembly and (ii) exploded view of the MCE and the soft grasper, devised as add‐on onto the rigid jaw (dVRK jaw) of a standard dVRK tool. The mechanochromic capability is provided by an SP‐doped silicone acting as MCE posed in between a transparent top plate and the fenestrated dVRK jaw, and laid down on a transparent base plate. For ease of development, the clutching pressure is transferred to indenters acting on the MCE, so that mechanochromic activation can be associated with a certain clutching pressure value. The surgeon is informed when reaching the aforementioned clutching level by the color change associated with mechanochromic activation.

We integrated an MCE on a previously developed soft grasper,^[^ ^22^ ^]^ in turn devised as add‐on onto a clinically used dVRK tool (Endowrist Cadiere Forceps, by Intuitive Surg. Inc., CA, USA). The idea was to provide a visible cue (based on color change) to the operator when reaching a predefined clutching pressure threshold, devised for practical clinical applications by considering tissue damage thresholds. Indeed, considering that laparoscopic surgical graspers are systematically in the visual field of the operator during minimally invasive surgery, color change of the mechanochromic effector can help avoid tissue damage (**Figure** **1**a).

At the material level, for implementing the MCE we chose a soft SP‐doped biocompatible polysiloxane, illustrated in Figure 1b(i) where a macroscopic mechanical indentation can return a colored region. At the molecular level, the SP acted as a crosslinker in the elastomeric network, and a macroscopic stretching of the polymer chains can result into the break of the spiro C─O bond and the transformation from the colorless SP to the colored MC. We synthesized the SP molecule containing two vinyl functional groups, one attached at the benzopyran ring, and one at the indoline ring at the N‐position,^[^ ^47^, ^58^ ^]^ as shown in Figure 1b(i). The functionalization of the SP monomer with crosslinking sites on the opposite site of the indole and benzopyran junction was largely investigated in SP derivatives. Such functionalization results in the lowest critical activation force and faster de‐/coloration rate.^[^ ^47^, ^58^, ^59^ ^]^ Furthermore, we optically characterized the molecule photochromism to understand how to foster the MC‐to‐SP kinetics, in order to reduce chromogenic recovery time and ring closing rate, thus enhancing the potential for application of the optomechanical switch close to realistic/clinical conditions.^[^ ^47^ ^]^ UV–vis absorbance spectra were collected for the different forms of the synthesized molecule (Figure 1b(ii)). A broad absorbance peak around 560 nm was observed in the MC spectrum, suggesting that green light (or in general white light) can foster the reversible SP formation, consistently with previous reports.^[^ ^60^ ^]^

At the tool level, we envisioned to integrate the MCE as sketched in Figure 1c(i). Specifically, we complemented the soft jaw grasper^[^ ^22^, ^23^ ^]^ with a mechanical transmission mapping the clutching pressure on the MCE through an indenter. When the clutching force overcomes the aforementioned predefined clutching threshold, mechanochromic activation occurs in correspondence of the indented area, thus resulting in a vision‐based DFS strategy helping the surgeon to respectfully manipulate tissue.

In the following, we report on the optomechanical characterization of SP‐doped elastomers (Section 2.1), by considering mechanochromic activation pressure and chromogenic recovery time for several elastomeric compositions. We then describe pressure transmission and MCE indentation strategies (Section 2.2), before reporting grasper demonstration in the dVRK (Section 2.3).

### 2.1 Optomechanical Characterization of SP‐Doped Elastomers

We characterized the SP‐doped elastomers as regards the mechanochromic activation pressure and the recovery time of the backward chemical reaction MC‐to‐SP.

#### 2.1.1 Mechanochromic Activation Pressure

**Figure 2:**
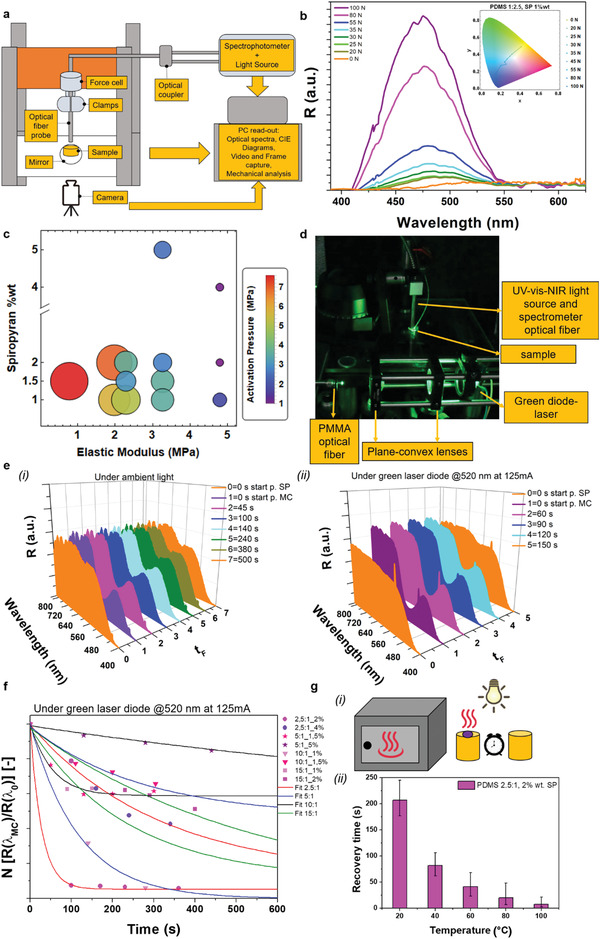
a) Custom‐made optomechanical setup. A universal testing machine was used, with the lower clamp substituted by a rigid transparent support. The SP‐doped elastomeric sample was laid down on the rigid constraint, and a highly reflective mirror in the visible range was mounted below. An optical fiber was mechanically clamped at the force cell connection. A UV–vis–NIR light source enlightened the sample to measure the colorimetric reflectance. The reflected light was decoupled in a spectrophotometer. Both mechanical and optical setup were connected to the PC‐readout, to collect optical spectra, CIE‐1931 diagram, mechanical force and displacement, as well as video and frame captures. b) Reflectance (*R*) spectra of the PDMS (2.5:1), 1% wt. SP‐doped sample detected by the optical fiber probe. Color legend describes the different spectra at fixed compression force detected by the universal testing machine. Spectra are normalized, defining the unit value at the dominant color of the SP‐doped elastomer (yellowish around 580 nm) not mechanochromically activated, and then highlighting the mechanochromic peak at around 480 nm. Increasing the indentation force, the changing coloration becomes more evident and entirely covers the indentation surface (Figure S2, Supporting Information). Inset: CIE‐1931 color diagram for the same sample. Every dot represents a triad of coordinates resulting in a macroscopic color detectable by the human eye. All the dots are connected to better visualize the blue shift. c) Mechanochromic activation pressure (averaged value per each sample with minimum *n* = 3 repetitions) bubble chart, based on polysiloxanes with several concentrations of SP‐doping. Polysiloxanes are identified by their elastic modulus determined in Table 1 (sample size *n* = 5, mean ± SD, curing temperature 90 °C, time curing 9 h). The size of the bubble radius and the color legend represent the activation pressure (extended data in Figure S4, Supporting Information, the asymmetric whiskers represent the largest and smallest values, i.e., range, for the *n* = 13 sample with at least three repetitions per each value). d) Optical test bench for time‐dependent MC‐to‐SP reaction in a black box. A green laser was supplied (DC power supply at 125 mA). The light was driven and the spot was collimated and reduced by means of two plane convex lenses into a PMMA‐optical fiber, and sent to the doped PDMS‐based samples. A UV–vis–NIR highly reflectance mirror was mounted below the transparent support holding the sample. The mirror spread the green light on the MC activated sample, and the optical fiber, which was connected to the UV–vis–NIR light source and to the spectrophotometer, collected the reflectance spectra. A digital timer counted the chromogenic recovery time. e) (i) Chromogenic recovery time of a PDMS (2.5:1), 2% wt. SP‐doped sample (cured at 90 °C per 9 h), under ambient light and at room temperature (25 °C), with *n* = 2 test repetitions. (ii) Chromogenic recovery time of a PDMS (2.5:1), 2% wt. SP‐doped sample (cured at 90 °C per 9 h), under green diode laser lighting and at room temperature (25 °C), with *n* = 2 test repetitions. f) Decay time characterization for all the SP‐doped PDMS samples (same optical bench as above). Time trend of the reflectance peak value at *λ*_MC_ = 480 nm, normalized by the value at the dominant wavelength (*λ*_0_ around 580 nm for all the composition except for the PDMS 15:1 about 650 nm). Data reported are averaged on *n* = 2 samples (error bars are not reported for ease of visualization). g) (i) PDMS 2.5:1 with 2% wt. SP‐doping (cured at 90 °C per 9 h) was heated at different temperatures through an oven (from 20 up to 100 °C). (ii) Corresponding chromogenic recovery times. The reported values on the bar chart are representative of the mean value on *n* = 5 test repetitions for each temperature. The asymmetric whiskers represent the range defined as the difference between the largest and smallest observations (Table S1, Supporting Information).

We tested the optomechanical properties of a series of PDMS (prepolymer/curing agent weight ratios: 2.5:1, 5:1, 10:1, 15:1), and Dragonskin20 (prepolymer/curing agent weight ratio: 1:1) round‐shaped samples, doped with different SP amounts (1.0%, 1.5%, 2.0%, 4.0%, and 5.0% w/w) and cured at 90 ± 5 °C for 9 h. We investigated the mechanochromic response of the selected doped elastomers under a rising mechanical compression, to find the activation pressure when a changing coloration arose. We realized an ad hoc optomechanical setup (**Figure** **2**a), designing the fiber optic as both mechanical indenter and optical probe, to record the compression force and the spectroscopic data. Compression force values were recorded from values applied at the natural color of the material (steady state), to the most visible blue/violet coloration. The optomechanical assembly was directly connected to the PC‐readout that can analyze reflectance optical spectra, and relative CIE‐1931 color space diagrams at varying indenting forces. We collected all the reflectance optical spectra at different indenting forces, for all our samples (Figure S1, Supporting Information). Figure 2b reported, as an example, the reflectance spectrum (*R*) for PDMS (2.5:1), 1% wt. SP‐doped. The optical properties did not change significantly when SP was dispersed in polymeric matrices of different nature (such as PDMS, and Dragonskin20). The inset in Figure 2b showed the chromatic behavior of the PDMS (2.5:1), 1% wt. SP‐doped sample. The sample hypsochromism shift was directly related to the rising mechanical force applied.

Once all the spectroscopic measurements were collected by varying the indentation forces, we investigated the correlation between the mechanochromic behavior of the polysiloxane samples and its mechanical properties, where each prepolymer/curing agent weight ratios was identified by the relative elastic modulus. Elastic moduli were calculated by monoaxial stress–strain traction tests on standardized dog‐bone samples not doped with SP (**Table** **1**). Small amounts of SP were demonstrated to not affect the mechanical properties of bulk polysiloxanes.^[^ ^47^ ^]^ Dog‐bone samples were cured at the same temperature of SP‐doped samples, to avoid affecting mechanical properties of the silicone matrix at different curing temperatures (even shown in Table 1).^[^ ^61^ ^]^ Furthermore, the mechanical performances of PDMS is affected by different percentage of the crosslinking sites, and it was proved that the more crosslinking sites are, the higher material elastic modulus results (Figure S3, Supporting Information).^[^ ^62^ ^]^ Figure 2c showed that slighter forces in “tougher silicones” are needed to pull/squeeze the disordered polymeric chain‐length distribution enabling the spiro C─O bonding scission, respect to lesser crosslinked polymers. This result was in line with the model developed by Wang et al.,^[^ ^63^ ^]^ asserting that the extent of MC activation monotonically decreases with the number of links in a network. Moreover, we did not detect substantial difference in minimum activation pressure in the range of doping from 2% to 4–5% (Figure 2c). Nonetheless, we observed that thermally postcuring the elastomers (with and without SP loading), caused the elastic modulus to considerably increase (Table 1), thus resulting in more brittle samples.^[^ ^64^ ^]^ However, the rate opening of the spiro C─O bond in the matrix was not affected by the annealing and/or postannealing; rather, it was mostly driven by the collective mechanical properties of the host matrix (Figure S13, Supporting Information). Thus, while increasing the elastic modulus of the material could effectively lower the activation pressure, it would also make the device more brittle and prone to irreparable damage. In this sense, we believe that the choice of a matrix with reasonable elasticity and rupture strain (%) is more suitable for the proposed application. We mainly focused on PDMS instead of Dragonskin20 because the latter needed a very high compression pressure to enable mechanochromism (out of the application scope). Then for simplicity of visualization, only Dragonskin20 (1:1), 1.5% wt. SP‐doped was reported. The PDMS (2.5:1), 2% wt. SP‐doped (cured at 90 °C per 9 h) provided the lowest activation pressure, namely, around 1.17 MPa (corresponding to average value of 38.8 ± 8.2 N for an indenter with a surface of 33.16 mm^2^). The aforementioned composition was thus regarded to as optimal in the present study, since the activation pressure is a key parameter to design the indenting structures in relief aimed to activate the MCE embedded in the sensorized soft effector (and a lower activation pressure facilitates such a design). Hence to embody the devised mechanochromic surgical grasper, an indenter‐induced pressure around 1.17 MPa must correspond to a chosen maximum tissue clutching pressure, i.e., to the maximum pressure threshold for the chosen tissue.

**Table 1:** The different polysiloxane‐based matrices mechanically tested using a universal testing machine (sample size *n* = 5, mean ± SD)

Material type	Prepolymer/curing agent weight ratios	Curing temperature (first polymerization/second polymerization) [°C]	Curing time (first polymerization/second polymerization) [h]	Elastic modulus [MPa]	Standard deviation [MPa]
PDMS	1.5:1	150/–	0.5/–	2.22	0.22
PDMS	1.5:1	90/150	0.5/5	4.47	0.26
PDMS	2.0:1	150/–	0.5/–	1.99	0.45
PDMS	2.5:1	90/–	9/–	4.79	1.18
PDMS	2.5:1	90/150	0.5/5	4.44	0.12
PDMS	2.5:1	120/150	0.5/5	6.20	0.18
PDMS	2.5:1	150/150	2/5	21.93	1.44
PDMS	5:1	90/–	9/–	3.26	0.10
PDMS	10:1	90/–	9/–	2.29	0.04
PDMS	15:1	90/–	9/–	1.97	0.16
PDMS	40:1	90/–	9/–	0.54	0.17
Dragonskin‐20	1:1	90/–	9/–	0.78	0.24

#### 2.1.2 Mechanochromic Activation Pressure at Different Temperatures

We performed experimental measurements to analyze the effect of temperature on the minimum mechanochromic activation pressure. The analysis was carried out considering the MCE‐DFS subjected to thermal annealing during mechanical indentation. We modified the optomechanical setup laying down the SP‐doped MCE on a hot‐plate controlling the temperature of the sample. We investigated a sample for each family of PDMS (2.5:1, 4% wt. SP‐doped cured at 90 °C; 5:1, 1.5% wt. SP‐doped cured at 90 °C; and 10:1, 1% wt. SP‐doped cured at 90 °C), heating them at different superficial temperature (25, 60, and 100 °C). Figure S12 of the Supporting Information shows no substantial differences between 25 and 60 °C at the same indentation force, encouraging the adoption of the developed MCE sensor device in the human body. Indeed, the collective thermal movement of the polymeric chain‐system does not modify the mechanical threshold required to open the spiro C─O bond, thus resulting in a weak dependence of the macroscopic output versus temperature for the considered mechanochromic pressure sensor.

#### 2.1.3 Chromogenic Recovery Time at Room Temperature

We realized a custom‐made optical bench setup under ambient light and under external optical pumping to analyze the recovery time of the backward chemical reaction MC‐to‐SP. Figure 2d reported the optical bench at room temperature (25 °C). The MC form was mechanically indented up to mechanochromic activation, and the spectroscopic data were recorded. In Figure 2e, we analyzed the reflectance spectra at varying time frames (*t*_F_), for the previously identified optimal SP‐elastomer concentration (PDMS 2.5:1, 2% wt. SP‐doping, cured at 90 °C per 9 h). In Figure 2e(i), the entire spectrum was recorded under ambient light. The plot showed the different timing frames until the complete recovery of the MC‐to‐SP form. A clear broad peak at around 480 nm was detectable at the starting point (at *t*_F_ = 1 = *t*_F0, MC_) of the MC form. The peak vanished after about 240 s, when the reflectance spectrum (at *t*_F_ = 5) was the equivalent than the SP colorless starting point (at *t*_F_ = 0 = *t*_F0, SP_). Figure 2e(ii), quantified the time reduction of the MC‐to‐SP chemical reaction once optically pumped with a green laser diode (*λ*_emission_ = 520 nm). Therefore, green light pumping (520 nm optical laser diode pump driven at 125 mA) allowed reducing the recovery time down to ≈150 s. In Figure 2f, we investigated the decay time for all the mechanochromic activated SP‐doped PDMS compounds (at varying prepolymer/curing agent composition, and % wt. doping) subjected to the same green laser diode optical pumping. We plotted the normalized ratio value (*N* [*R*(*λ* = 480 nm)/*R*(*λ*_0_)]), in function of time (*t*). *R*(*λ* = 480 nm) represented the MC reflectance peak at 480 nm, and *R*(*λ*_0_) (where *λ*_0_ is equivalent to 580 nm for all the prepolymer/curing agent composition except for the 15:1 that shows a broad peak at 650 nm) was the reflectance peak at the dominant yellowish/reddish sample coloration. Based on the decreasing time trend shown in the figure at hand, the recovery time of the optimal SP‐doping (PDMS 2.5:1, 2% wt. SP‐doping, cured at 90 °C per 9 h) resulted to be shorter than that one of different concentrations, thus further supporting the motivation to use the aforementioned concentration for developing the proposed DFS‐MCE. More in general, we experimentally proved that a percentage of doping ranging from 1% to 2% lead to a shorter recovery time than higher percentage of doping (e.g., 4–5%). Several variables might influence the ring‐closing kinetics and we did not perform a mechanistic investigation in this regard. For example, it is known that, in analogue systems, in the absence of visible light, the ring closing kinetics of SP is as well a function of the glass transition temperature.^[^ ^65^ ^]^ Let us mentioned that, by reducing the thickness of the active material, it may be possible to further shorten the recovery time by means of having less chromophores in the addressed volume.^[^ ^66^ ^]^ The obtained recovery time at room temperature is not yet low enough to enable real‐time dynamic sample manipulation, yet its timescale seems not to prevent the use of the proposed sensing strategy for the envisioned grasping and retraction tasks. In order to refine this consideration in view of effective surgical applications, we then assessed the effect of body temperature on the recovery time.

#### 2.1.4 Chromogenic Recovery Time at Body Temperature

In view of the potential translation of the proposed technology to the biomedical field, we also evaluated the recovery time of the thermally activated backward reaction MC‐to‐SP. We heated the optimized composition (PDMS 2.5:1, 2% wt. SP‐doping, cured at 90 °C per 9 h) in an oven and after taking it out, we adopted an indenter (radius 1.23 mm, and around 24 N compression force), in order to obtain a clearly visible chromogenic region (Figure 2g(i)). Figure 2g(ii) showed the recovery time decay trend at increasing temperature. In particular, when passing from 20 to 40 °C, the recovery time reduced by nearly three times. This supports the potential application of the proposed mechanocromism‐based grasping technology to medical interventions in the human body. At the molecular scale, a higher temperature increases the rate of closure of the spiro C─O chemical bond that corresponds to the most thermodynamically stable SP conformation.^[^ ^67^, ^68^ ^]^ Then, the superposition of effects given by light and heat may speed up the rate of the backward reaction MC‐to‐SP, approaching typical thresholds of electronic sensors.

### 2.2 Integration of the Mechanochromic Material in the Sensorized Grasper

**Figure 3:**
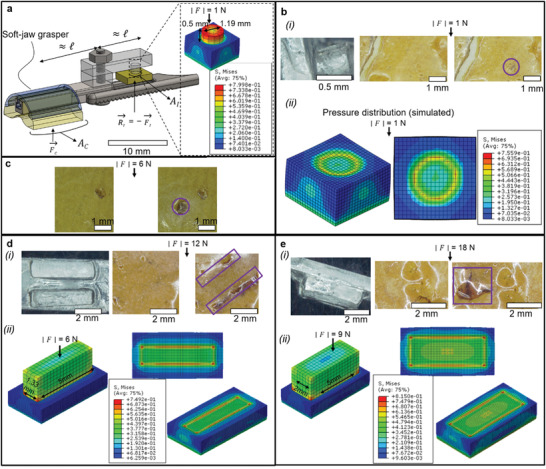
a) Schematic of the pressure transmission elements mapping the clutching pressure to the indentation pressure also based on the corresponding clutching areas. b) (i) MCE indentation (cylindrical indenter) at 1 N: (left) optical microscope image of the indenter, (middle) sample prior to compression, and (right) sample after compression (mechanochromic activation highlighted through the circle). (ii) Complementary stress distribution obtained by numerical simulations. c) MCE indentation (same cylindrical indenter as above) at 6 N, (left) prior and (right) after compression (mechanochromic activation highlighted through the circle). d) (i) MCE indentation (two parallelepipedal indenters) at 12 N: (left) optical microscope image of the indenter, (middle) sample prior to compression, and (right) sample after compression (mechanochromic activation highlighted through the rectangle). (ii) Complementary stress distribution obtained by numerical simulations. e) (i) MCE indentation (two parallelepipedal indenters) at 18 N: (left) optical microscope image of the indenter, (middle) sample prior to compression, and (right) sample after compression (mechanochromic activation highlighted through the rectangle). (ii) Complementary stress distribution obtained by numerical simulations.

We integrated the MCE into the soft‐jaw grasper as sketched in **Figure** **3**a, namely by means of a sort of fulcrum transmission transferring the clutching force *F*_c_ (acting on the soft jaw) into an indentation force *F*_i_ (acting on the MCE). For simplicity, we assumed the lever arm to be of comparable length (labeled by l in Figure 3a) for the aforementioned mechanical actions, so that *F*_c_≅*F*_i_ and the clutching pressure *p*_c_ (acting on the corresponding jaw area *A*_c_) can be immediately linked to the indentation pressure *p*_i_ (acting on the corresponding indenter area *A*_i_) as follows: *p*_i_≅ *p*_c_(*A*_c_/*A*_i_). The latter relation permits to regard the *A*_c_/*A*_i_ as “geometrical amplification factor” to be determined for mapping a working pressure *p*_c_, into a pressure *p*_i_ suitably above the mechanochromic activation threshold. In a clinical perspective, the clutching pressure should be kept below a tissues‐specific damage threshold^[^ ^76^, ^77^, ^78^ ^]^ (see **Table** **2**).

**Table 2:** Tissue damage threshold for porcine organs

Tissue type	Damage threshold [kPa]	Reference
Porcine ureter	180	^[^ ^76^ ^]^
Porcine small bowel	240	^[^ ^76^ ^]^
Porcine liver	180	^[^ ^76^ ^]^
Porcine liver	162.5 ± 27.5	^[^ ^77^ ^]^
Porcine liver	146	^[^ ^78^ ^]^

First, we considered the cylindrical indenter shown in Figure 3a, whose *A*_i_ leads to a mechanochromic activation (1.17 MPa for PDMS 2.5:1, 2% wt. SP‐doped, cured at 90 °C per 9 h) for *F*_c_ around 1 N (Figure 3b(i)). The corresponding mechanochromic footprint at 6 N clutching force is more pronounced, as shown in Figure 3c. Moreover, higher clutching forces could be mapped to multiple indenters, as shown in Figure 3d(i),e(i), where *A*_i_ is defined so as to reach the mechanochromic activation (as above) for 12 and 18 N clutching force, respectively (please, notice that in such a case *F*_c_≅2*F*_i_). The latter value, in particular, was considered because it is the maximum clutching force for which the adopted soft jaw grasper was designed.^[^ ^22^ ^]^ As a complementary information, in Figure 3b(ii),d(ii),e(ii), we also reported the von Mises stress obtained by numerical simulations, with the aim to illustrate the spatial distribution of the indentation stress (featuring some concentration close to the indenter edges, as well reported in literature). Figure 3b–e was optical microscope images reporting the activated mechanochromic area.

### 2.3 Mechanochromic Grasper Demonstration on the da Vinci Research KIT

**Figure 4:**
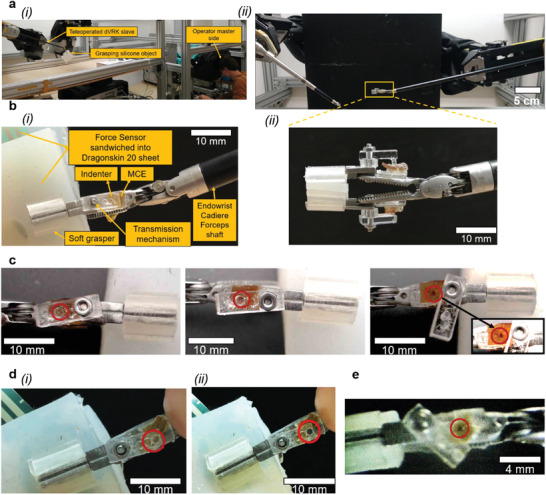
a) The dVRK platform used for the final grasper demonstration: (i) master side and (ii) slave side. b) Mechanochromic grasper, implemented as add‐on onto a dVRK tool (Endowrist Cadiere Forceps), clutching a soft sample: (i) key components, (ii) side view highlighting the pressure transmission elements. c) Teleoperated mechanochromic grasper clutching a soft sample at 5 N: (left) prior to mechanochromic activation, (middle) after mechanochromic activation, (right) activated spot highlighted by removing the covering Plexiglas plate. d) Mechanochromic grasper clutching a soft sample at 7 N (by directly pulling tool cables): (i) prior to mechanochromic activation and (ii) after mechanochromic activation. The force sensor, which was sandwiched between the grasped soft sheets to simultaneously measure the clutching force, is visible in the upper‐left figure region. e) Endoscopic image of the activated mechanochromic grasper, as acquired through the endoscopic camera natively integrated in the dVRK platform.

We finally integrated the mechanochromic grasper into the dVRK slave, to be teleoperated by the master console (**Figure** **4**a(i),(ii)). The available dVRK surgical robotics platform permits to clutch with forces up to nearly 6 N: we thus implemented the pressure transmission strategy onto the MCE in the dVRK tool by using the cylindrical indenter introduced in Figure 3b. More specifically, we integrated the MCE/transmission onto a dVRK surgical tool (Endowrist Cadiere Forceps, by Intuitive Surg. Inc., CA, USA) as add‐on (Figure 4b(i),(ii)), for ease of development. Using the teleoperated mechanochromic grasper, we grasped a soft material sample, as shown in Figure 4c: 5 N clutching force (measured through a thin sensor framed in the grasped sample) activated the mechanochromic response, as shown by the bluish spot, also shown by removing the Plexiglas plate for ease of rendering. The considered clutching force is clinically representative for some surgical procedures^[^ ^53^, ^79^, ^80^, ^81^, ^82^, ^83^, ^84^ ^]^ (**Table** **3**). Higher clutching forces can enhance the mechanochromic response, as shown in Figure 4d(i),(ii), where 7 N clutching was enforced by directly pulling the Endowrist Cadiere Forceps tensioning cable. Finally, to demonstrate the potential for translation toward a real surgical scenario, we also acquired some pictures of the mechanochromic response through the endoscopic camera natively integrated in our dVRK platform (Figure 4e). A complementary video of the dVRK implementation is available in Video S1 of the Supporting Information. For completeness and considering tissue holding for surgical retraction, we also assessed that the induced mechanochromic response was stable (i.e., without color fading) over a 10 min clamping. Let us observe that we deliberately aimed to use the dVRK to demonstrate the potential for (pre‐)clinical translation. However, considering that the minimum MCE activation pressure associated with the considered experimental range of SP‐doping was sensibly higher than typical pressure thresholds for tissue damage (see Table 2), we had to also introduce a pressure transmission strategy when modifying the dVRK base tool. Moreover, considering that the used dVRK platform was a first‐generation system, we could not exert clutching forces as high as those available on current da Vinci clinical systems (e.g., da Vinci S, Si, and Xi), nor we could benefit from higher resolution currently available for its endoscopic camera.^[^ ^69^ ^]^ Nonetheless, the obtained results demonstrate the possibility to integrate a mechanochromic grasper in a state‐of‐the‐art surgical robotic system, by leveraging material intelligence to achieve sensing capabilities without resorting to extremely complex electronics implementations.

**Table 3:** Minimum retraction force for different organs and the relative minimum needed grasping force

Organ	Surgery	Retraction force (N), weight (g)	Grasping force (N) *μ* = 0.6	Grasping force (N) *μ* = 0.9	Reference
Gallbladder	Abdominal intervention	1 N, (≈100 g)	1.67	1.11	^[^ ^53^ ^]^
Stomach	NA	1.25–1.75 N, (≈125–175 g)	2.08–2.92	1.39–1.94	^[^ ^79^ ^]^
Pancreas	NA	0.4–1.8 N, (≈40.9–182 g)	0.67–3	0.44–2	^[^ ^80^ ^]^
Uterus	Uterus removal	5–16.9 N, (≈500–1690 g)	8.33–28.17	5.56–18.78	^[^ ^81^ ^]^
Gallbladder + liver	NOTES	6.63 N, (≈663 g)	11.05	7.37	^[^ ^82^ ^]^
Pig colon	Colon surgery	5 N, (≈500 g)	8.33	5.56	^[^ ^83^ ^]^
Porcine liver	Exposure of gallbladder	13–21 N, (≈1310–2100 g)	21.67–35	14.44–23.33	^[^ ^84^ ^]^

## 3 Conclusion

**Figure 5:**
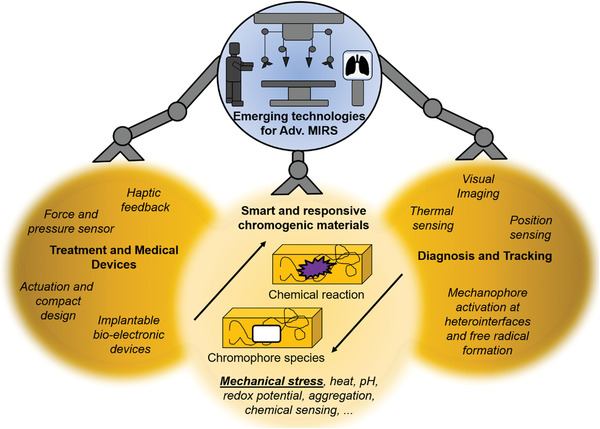
Schematic outlook to the potential extended use of chromophores in minimally invasive surgery, and in particular for robotic surgery (MIRS), for both diagnostic and interventional tasks. The present study, indeed, provides the first application of mechanocromism in MIRS, yet the demonstrated integration of such responsive materials in miniature tools can pave the way for further tools and applications.

We characterized, realized, and preliminary validated a mechanochromic grasper capable to provide a visual feedback for safe tissue manipulation during mininvasive surgery, as illustrated through grasper integration into the dVRK surgical robotics platform. The sensing core of the grasper is an SP‐doped MCE, which was characterized in terms of mechanochromic activation pressure and chromogenic recovery time, also considering body temperature in view of the targeted surgical application. The proposed mechanochromic sensing element was achieved by integrating switchable and reversible active chromophores in an elastomeric matrix, thus obtaining an electronics‐free sensing system that, beyond the addressed surgical realm, could inspire innovative solutions for, e.g., adaptive displays, optoelectronics, biomedical luminescent and color‐changing devices, soft robotics, and dynamic camouflage skins.^[^ ^70^, ^71^ ^]^ Minimally invasive surgery, however, can strongly benefit by also drawing from material science and chemistry, and in particular from the use of mechanophores,^[^ ^72^ ^]^ for both diagnostic and interventional tasks, as hinted in **Figure** **5**. For instance, the integration of such responsive materials can be used for providing further feedbacks to the operator, for simplifying tool design and foster miniaturization based on passive properties, to enrich diagnostic capabilities also for micro/nanoscale agents possibly reacting to specific/selective environmental conditions.^[^ ^73^ ^]^ The proposed SP‐doping of PDMS is expected not to be harmful for the human body;^[^ ^74^ ^]^ however, for enacting the above vision, further investigations on biocompatibility should be carried out. Furthermore, more refined embodiments should be studied:, e.g., in relation to the addressed surgical application, direct integration of the sensing material into the soft jaws should be pursued, also in view of more refined usability assessment (including, e.g., sterilization, industrial packaging, etc.). Moreover, further investigations on the combination of illumination and temperature effects are needed for reaching real‐time chromogenic recovery times, i.e., to speed‐up the backward MC‐to‐SP reaction, as needed for dynamic manipulation tasks. In addition, multicolor and tunable colorimetric information from different polymers doped with different % wt. of SP could be studied for spatially resolved or multiple‐activation‐threshold applications. To the best of our knowledge, in spite of the limitations associated with the proposed study, our achievements provide the first application of mechanochromic materials in surgery. Indeed, we took a first step toward the use of such responsive materials for developing electronics‐free miniaturized tools, with potential for application also beyond the biomedical sector.

## 4 Experimental Section

### Chemical Synthesis and Sample Fabrication

All the reagents were purchased from Sigma‐Aldrich or Fisher Scientific and used without further purification. All the samples were fabricated by crosslinking PDMS (Sylgard 184), and Dragonskin20 (Smooth‐On) with the SP bifunctional monomer containing two vinyl groups (Figure S5(SP), Supporting Information). Details of the chemical synthesis and sample fabrication can be found elsewhere,^[^ ^48^ ^]^ and for the sake of clarity are resumed in Figure S5 of the Supporting Information. Column chromatography the hydroxyl‐functionalized SP (Figure S5(3), Supporting Information) was purified, and the procedure by Gossweiler et al.^[^ ^59^ ^]^ was followed for the synthesis and purification of the final product (Figure S5(SP), Supporting Information). However, in this case, the final product was further purified via column chromatography (neutral Al_2_O_3_, dicloromethane). All the structures of the chemical compounds were verified by ^1^H‐NMR and ^13^C‐NMR (Bruker 400 MHz spectrometer at 300 K, Figure S6–S9, Supporting Information). To prepare the doped samples, the appropriate amount of bifunctional SP monomer was dissolved in 1.2 mL of DCM in a glass vial together with the prepolymer (PDMS or Dragonskin20), and stirred vigorously for 1 h. All the weight fractions (prepolymer/curing agent weight ratio 2.5:1, 5:1, 10:1, 15:1, 1:1) were designed in order to replicate sample with SP weight percentages of 1.0%, 1.5%, 2.0%, 4.0%, and 5.0%. After 1 h, the curing agent was added to the mixture and stirred for an additional hour. The solution was then poured into a rounded mold and the solvent removed under vacuum (200 mbar). Finally, the mold was placed in an oven at 90 ± 5 °C until cured (typically 9 h). The color of mechanochromic samples varied from a slight yellow to a dark‐orange coloration.

### Mechanical Material Characterization

Dog‐bone standardized shaped tester of PDMS (2.5:1, 5:1, 10:1, 15:1, 40:1) and Dragonskin 20 (1:1) were cured into a tailored mold at 90 ± 5 °C until cured per 9 h. These samples are not SP‐doped, and were fabricated with the same temperature and curing time of the corresponding SP‐doped PDMS samples analyzed in Figure 2. Further dog‐bone shaped tester of PDMS (1.5:1, 2.0:1, 2.5:1) cured at different temperature (90, 120, and 150 °C) with a first polymerization procedure, and a second postannealing (150 °C), varying the curing time (0.5, 2, 5 h), were evaluated as reported in Table 1. The Zwick/Roell Z005 universal testing machine (UTM) traction tests were repeated at least five times per each sample, and in the elastic deformation range the elastic modulus was deducted. Standard deviation values are reported for all the measurements.

### Optomechanical Setup

The custom‐made optomechanical setup was designed to detect mechanical deformation and spectroscopic information on the SP‐doped samples. The size (20.4 mm ± 0.1 mm diameter), thickness (4.0 mm ± 0.5 mm), and geometry of the SP‐doped samples were standardized, and calibrated in order to support and dampen the mechanical indentation displacement without fracture. The UTM was used for mechanical measurements and as a linear trail. A silica‐based fiber optic (radius probe = 3.25 mm ± 0.01 mm) was clamped to a 1 kN force cell (Zwick/Roell Xforce P) and it acted as both mechanical and optical probe to detect reflected wavelengths on the surface of the samples. An optical coupler split the fiber respectively to the UV–vis–NIR light source and spectrophotometer (Micropack Ocean Optics NanoCalc‐XR). The entire optomechanical setup was covered with a dark cloth to reduce the environmental light noise into the optical fiber. When the optomechanical probe compressed the sample the MC activation resulted in a ringed violet/dark‐blue coloration through a full circular spot at higher indentation forces. Mechanical indentation triggered the 6‐*π* electrocyclic ring‐opening reaction giving rise to the characteristic coloration of the MC form. The adopted experimental protocol required that the optomechanical probe approached the sample at 0.5 mm min^−1^, compressed it until a predetermined force value, then it released the compression phase to allow the elastomeric matrix to dampen the pressure, and it collected optical data with a predetermined optical spectroscopic integration time (lesser than the MC‐to‐SP recovery time) on a flattened surface. In this way, the temporal automated system allowed the optical signal integration after the compression and the elastomeric relaxation time‐phase (a steady‐state customized protocol). The compression force range was, e.g., for PDMS 2.5:1, from 0 to 80 N, while for PDMS 15:1 from 0 to 250 N. A control on the indentation displacement into the sample thicknesses followed the measurement, in order not to acquire the rigid support constraint force. Indeed, the sample was laid down on a Plexiglas transparent support, and behind it, an UV–vis–IR high reflective plane metallic mirror was fixed to optimize the collection of all the reflected light while the probe was pushing the sample, and to perform optical acquisition with the ease of a web‐camera directed on the sample to control macroscopic polymeric fractures. Optical pictures of the indented sample (e.g., PDMS 2.5:1, 1% wt. SP‐doping) were reported in Figure S2 of the Supporting Information. Then the PC‐readout with the SpectraSuite software and the UTM software collected and controlled the optomechanical acquisition. The reflective‐mode measurements were acquired in a 2° process, with f8 light source as reference. SpectraSuite software allowed to collect both the reflectance spectrum and the CIE‐1931 diagram for each force threshold chosen. For each sample a couple of indented regions were analyzed and returned almost the same value for spectroscopic measurements and color diagrams net of scattering surface issues depending on the sample fabrication procedure.

### Spectrophotometer Measurement

UV–vis–NIR absorption spectra were recorded with a PerkinElmer LAMBDA 45 spectrophotometer. For the measurements (270–1000 nm), 3 mg of SP in 2 mL DCM solutions were placed in optical glass macrocuvette (10 mm light path). The MC spectrum was recorded after the optical stimulation of the solution by means of an external UV light source.

### Mechanochromic Activation Pressure at Variable Temperature

The optomechanical setup was modified, laying down the samples doped with SP on an hot plate, substituting the support with the mirror with the thermal source. Three samples (2.5:1, 4% wt. SP‐doped cured at 90 °C; 5:1, 1.5% wt. SP‐doped cured at 90 °C; and 10:1, 1% wt. SP‐doped cured at 90 °C) were tested and heated at different superficial temperature (25, 60, and 100 °C). Per each sample at least two repetitions of colorimetric measurements were acquired, and the thermal annealing was prolonged for maximum 20 min, thus there are no substantial changes in the mechanical properties of the host matrix. The data reported in Figure S12 of the Supporting Information are carried out with average ± SD (Microsoft Office Excel 2016), and the spectral data are acquired with the same procedure reported in the Optomechanical Setup section.

### Chromogenic Recovery Time Measurement at Room Temperature

The series of PDMS % wt. SP‐doped samples were mechanically indented with a standardized indenter and a precision scale meter (Practum5101‐1S, Sartorius Lab), and the time‐dependent optical measurements reported the recovery time from the colored form to the colorless MC‐to‐SP. In a black box, the sample laid down on a transparent support and behind it there was an UV–vis–IR highly reflective plane metallic mirror. A silica‐based optical fiber pointed the sample. A drop of Immersol 518F immersion oil for fluorescence‐microscopy was in between the optical fiber and the sample. The optical fiber was split in two branches, to the UV–vis–NIR light source and spectrophotometer (Micropack Ocean Optics NanoCalc‐XR). It measured the reflectance spectra (*R*) at different time steps (*t*_F_), when the system was under environmental light or when a green light was pointed. Indeed, an optical bench tailored setup, presented a linear translator where a diode laser (PLT5 520 nm Osram OptoSemiconductor, *I*_F_ = 125 mA, optical output power 50 mW) was fixed. The laser was powered by a DC power supply through a constant current driving circuit at around 115–125 mA. Two plane‐convex lenses focused and controlled the spot dimension into a 0.5 mm PMMA plastic optical fiber, and the other termination was fixed by mean of a bare fiber terminator and connector, pointed at the highly reflective UV–vis–IR plane mirror. The green rays were driven through the sample MC‐activated spot (Figure S10, Supporting Information). The SpectraSuite software acquired the reflectance spectra, and with a digital timer, the recovery time under green light pumping and under ambient light was reported. The decay‐time trend for all the compositions was reported plotting the ratio between the *R*(*λ*_MC_)/*R*(*λ*_0_) at varying time only under green pumping. *λ*_MC_ was the peak of the MC molecular broad spectrum (peaked at around 480 nm), and *λ*_0_ was the dominant wavelength of the sample doped with SP closed form (yellowish at *λ*_0_ = 580 nm for all the PDMS composition except for 15:1 that appears reddish at *λ*_0_ = 650 nm).

### Chromogenic Recovery Time Measurement for Different Temperatures

The optimized PDMS (2.5:1), 2% wt. SP‐doped rounded sample was put in an oven at different temperatures (40, 60, 80 °C and 100 ± 5 °C) for 1 min for five times each. Once taking it out from the oven, it was laid down on a metal plate of a precision scale, and with a pointed stick (4.79 mm^2^) it was indented until generated a predetermined value of force (25 ± 1 N) measured with a scale meter to achieve a clearly visible mechanochromic spot. Then at standard ambient temperature (20–25 °C), the sample was left to cool down, and it was visually checked and recorded with a digital timer the recovery time when the mechanochromic blue/violet region faded.

### Mechanochromic Material Indentation

A 4 × 5 × 2 mm^3^ MCE brick (PDMS 2.5:1, 2% wt. SP‐doped), mechanically constrained through a transparent Plexiglas plate 4 × 5 × 2 mm^3^ as shown in Figure 3a, was considered. Cylindrical (diameter 1.19 mm, height 0.5 mm) and parallelepipedal Plexiglas indenters (1.33 × 5 × 2, and 2 × 5 × 2 mm^3^) were glued to the aforementioned plate. All the Plexiglas components were laser‐cut by a Versalaser machine (Universal Laser Systems Inc., AZ, USA). MCE compression was activated by applying a load simultaneously measured through an underneath precision scale meter. Pictures of the indented MCE were taken by using a digital optical microscope (Hirox Ltd., Japan) field of view. Complementary finite element analysis was performed by using Abaqus (Dassault Systèmes, Vélizy‐Villacoublay, France). In this regard, Plexiglas indenters were modeled as linearly elastic materials (elastic modulus 3 GPa, Poisson's ratio 0.37),^[^ ^75^ ^]^ whereas a Neo‐Hookean model was chosen for the MCE (with parameters C10 = 0.798 MPa and D1 = 0, as derived from the mechanical characterization of the 2.5:1 PDMS reported above). Standard 3D stress elements were adopted, as well as inviscid contact. Grid independence was checked.

### Grasper Demonstration on the da Vinci Research Kit

A dVRK platform was adopted for the final grasper demonstration. The dVRK is a research platform built upon the components of the first‐generation da Vinci Surgical System (Intuitive Surg. Inc., CA, USA). The dVRK features a teleoperative architecture, where a master console controls two slaves (besides the endoscopic camera arm). The dVRK tool selected for integrating the mechanochromic grasper was an Endowrist Cadiere Forceps (by Intuitive Surgical, product number 400049). Once integrated the mechanochromic sensing element through the cylindrical indenter transmission system, the as‐obtained end‐effector was teleoperated and a soft material sample composed of Dragonskin20 silicone sheets, also sandwhiching an FSR‐402 force sensor (Interlink Electronics, Inc., USA) for measuring the corresponding clutching force, was clutched. The force sensor was controlled by an ArduinoMega microcontroller, and it was previously calibrated with an indentation procedure on a precision scale. Pictures were taken by using both a smartphone camera (HD resolution 1080 p at 30 fps) and the endoscopic camera natively integrated into the available dVRK platform.

### Statistical Analysis

For mechanical material characterization *n* = 5 dog‐bone shaped samples were adopted. Table 1 reports the mean value ± SD, evaluated by means of Microsoft Office Excel 2016. For the optomechanical measurements *n* = 13 samples were tested, and for each sample at least three regions were spectroscopically investigated. Absorption spectra were acquired with SpectraSuite software, and the spectra in Figure S1 of the Supporting Information were plotted normalizing the reflection values at *λ*_0_ (dominant wavelength), in order to amplify the visibility of the reflection value at the mechanochromic/merocyanine wavelength (*R*(*λ*_MC_)). All the spectroscopic data were normalized (OriginPro 8.1, OriginLab). The ratio between *R*(*λ*_MC_) and *R*(*λ*_0_) at different indentation force values, was plotted for each SP‐doped PDMS composition and reported in Figure S11 of the Supporting Information. The squared dots represent the averaged values detected in at least three regions of the same sample. The averaged minimum mechanochromic activation force was obtained considering the mean value and the variance between three points. These data were confirmed by spectroscopic data (Figure S1, Supporting Information) and eyesight. The asymmetric whiskers shown in Figure S4, Supporting Information were evaluated considering the range (i.e., difference between the lowest and highest values) of the averaged *R*(*λ*_MC_)/*R*(*λ*_0_) = 1.15 (inferior limit) and *R*(*λ*_MC_)/*R*(*λ*_0_) = 1.5 (superior limit). As regard the mechanochromic activation pressure at variable temperature, *n* = 1 sample for each of the three families of PDMS prepolymer/curing agent weight ratio, was analyzed, and for each sample at least two acquisitions were carried out. The reported data in Figure S12 of the Supporting Information represent the averaged value ± SD (Microsoft Office Excel 2016), and the reflectance spectra are plotted with the aforementioned normalization procedure in the optomechanical measurement section (OriginPro 8.1, OriginLab). For the Chromogenic Recovery Time Measurement at Room Temperature section, the test repetitions for each sample was *n* = 2, and the reported points represent the averaged value ± SD (Microsoft Office Excel 2016, error bars in Figure 2f were not reported for ease of visualization). As regard the chromogenic recovery time measurement for different temperatures, the sample size was *n* = 5 for each temperature. A descriptive statistics analysis was performed evaluating the range, mean, standard deviation, and median (Table S1, Supporting Information, OriginPro 8.1, OriginLab).

## Conflict of Interest

The authors declare no conflict of interest.

## Supporting information

Supporting Information

Supplemental Video 1

## Data Availability

Research data are not shared.
