# Supplementary material for: Toward Mechanochromic Soft Material‐Based Visual Feedback for Electronics‐Free Surgical Effectors
Source: Adv Sci (Weinh). 2021 Jun 2;8(15):2100418. doi: 10.1002/advs.202100418 (PMC8336492; doi:10.1002/advs.202100418)
Supplement: Supplementary file 1 — Supporting Information [file ADVS-8-2100418-s001.pdf]

© 2021 Wiley-VCH GmbH

ADVANCED SCIENCE
Open Access

# Supporting Information

for *Adv. Sci.*, DOI: 10.1002/advs.202100418

# Toward mechanochromic soft material-based visual feedback for electronics-free surgical effectors

*Goffredo Giordano, Mariacristina Gagliardi, Yu Huan, Marco Carlotti, Andrea Mariani, Arianna Menciassi, Edoardo Sinibaldi, Barbara Mazzolai*

**SUPPORTING INFORMATION**

**Toward mechanochromic soft material-based visual feedback for electronics-free surgical effectors**

*Mariacristina Gagliardi, Yu Huan, Marco Carlotti, Andrea Mariani, Arianna Menciassi, and Goffredo Giordano*, Edoardo Sinibaldi*, Barbara Mazzolai**

**Video SI 1** The video first shows mechanochromic grasper, as obtained by integrating a 2 mm thick SP-doped polymer as sensing element into a da Vinci Surgical tool (Endowrist Cadiere Forceps by Intuitive Surgical). The mechanochromic equipment is actuated providing 7 N clutching force, while grasping a silicone tube and a Dragonskin20 sheet. Color change due to mechanochromic activation is highlighed. Then, integration of the mechanochromic soft grasper into the da Vinci Research Kit (dVRK) platform is demonstrated. Specifically, master-slave operations are shown, for grasping the aforementioned sheet at 5 N clutching force, still highlighting mechanochromic activation; the transparent plate included in the transmission mechanism is also removed, for ease of visibility. (Once clutching is removed, all the shown colored spots recover the original color, with a characteristic time determined by the chromogenic recovery time).

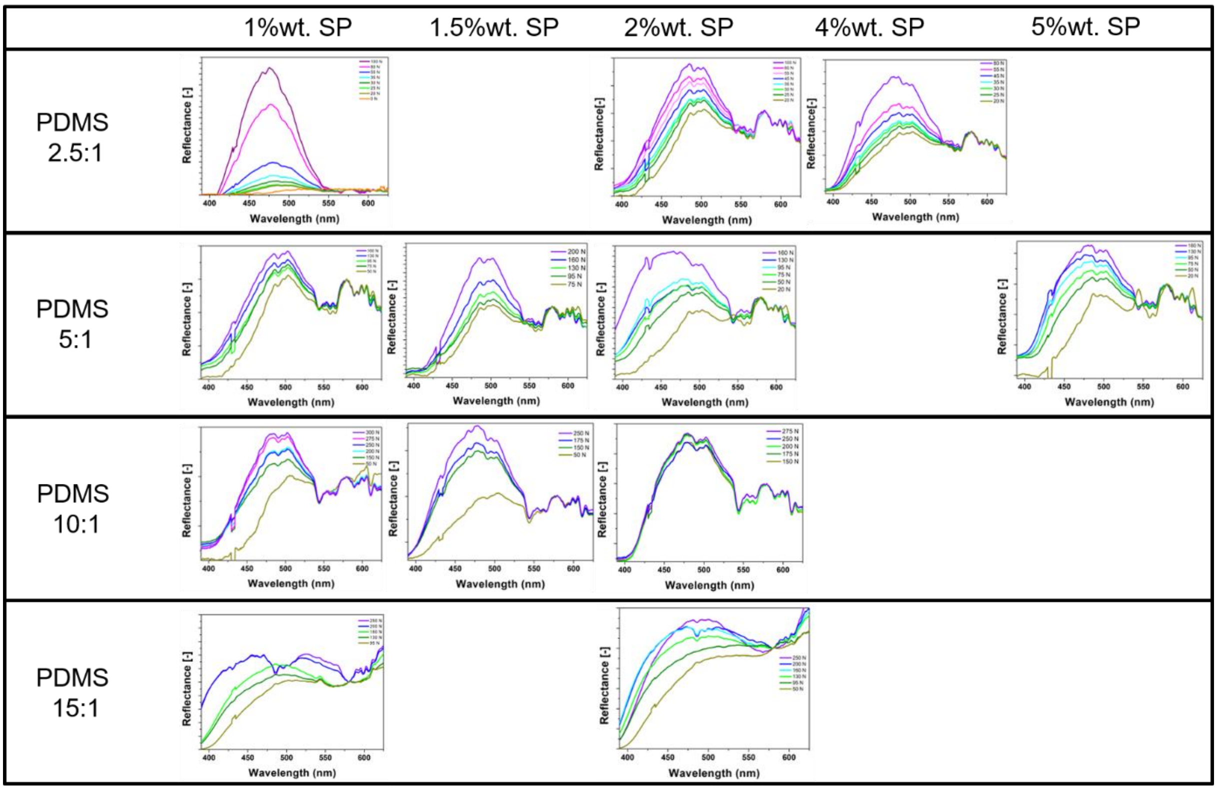


**Figure SI 1** All the reflectance spectra derived by the setup in **Figure 2a** with PDMS at different prepolymer/curing agent weight ratio composition and different percentage of SP-doping. The optical range plotted is between 390 nm and 625 nm to report the dominant wavelength peaked at 580 nm for all the composition except for the 15:1 that shows a broad peak at 650 nm. All the color legend return the indentation forces at which the spectroscopic measurements are acquired. For Dragonskin 20 the spectra are not reported because the minimum chromogenic indentation force is on the order of 200 N.

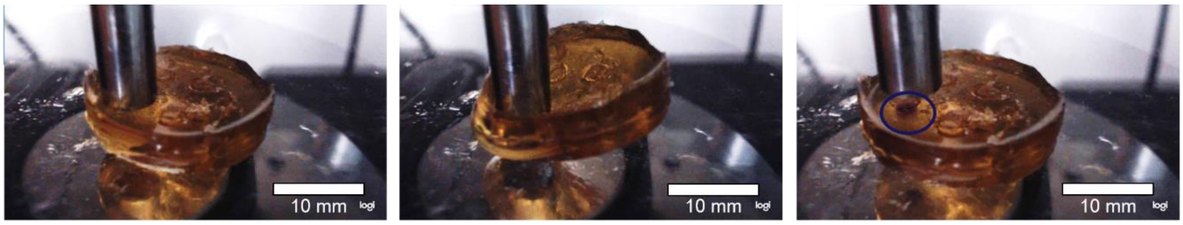


**Figure SI 2** PDMS 2.5:1 1% wt. SP-doping, with indentation force of 80 N. Blue circle guide the reader to the changing coloration probe footprint.

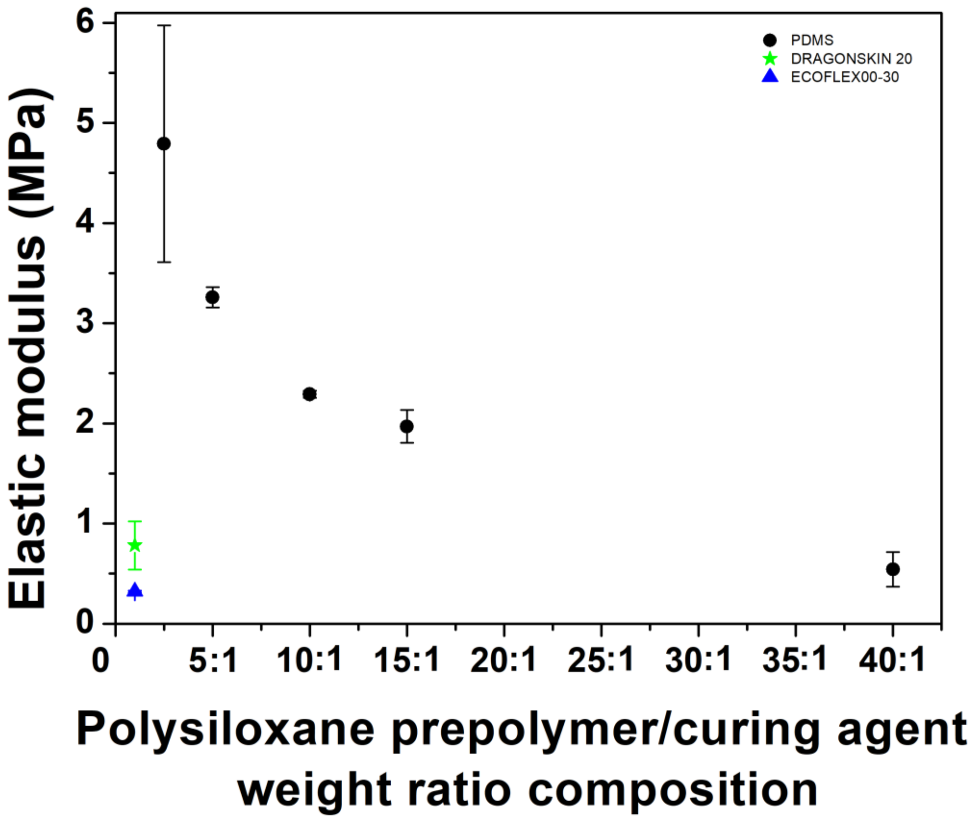


**Figure SI 3** Elastic modulus vs. polysiloxane prepolymer/curing agent weight ratio composition. All the data are acquired at a pre-determined curing temperature (avg. 90 °C ± 5 °C) for dog-bone samples for 9 h (please refer to **Table 1**, n=5, mean ± SD). Black circle represents PDMS. Green star stands for Dragonskin20 and blue triangle is for Ecoflex 00-30. Error bars represent the standard deviation for a set of five measurements for each point.

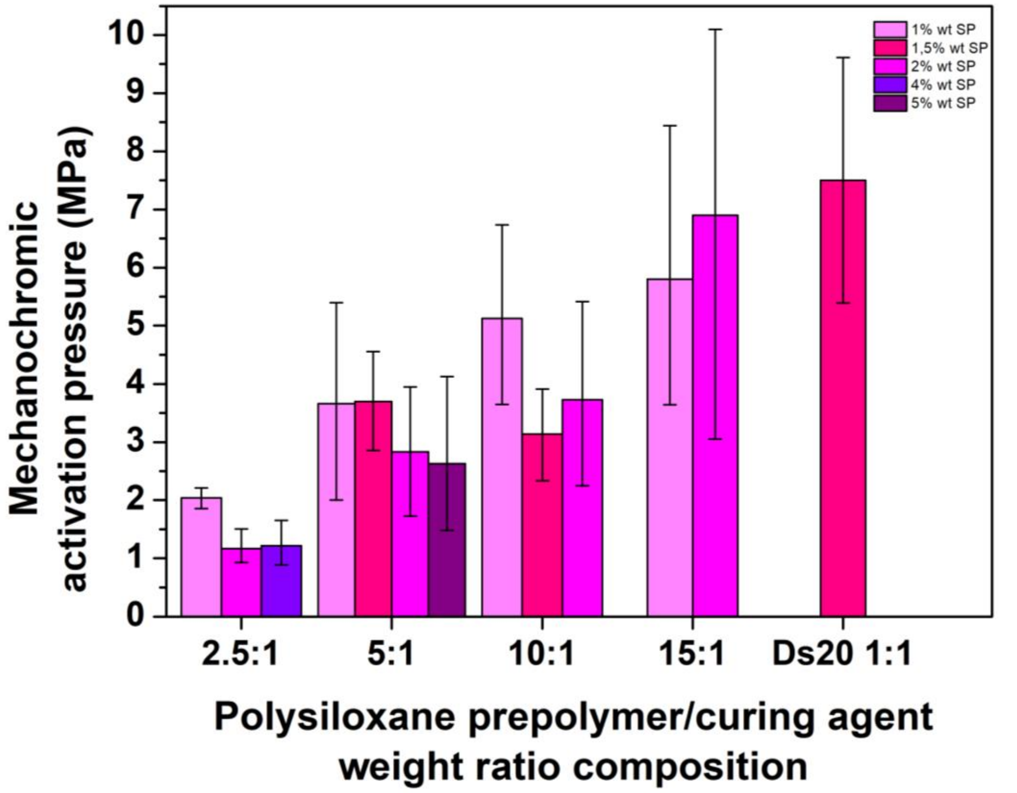


**Figure SI 4** Mechanochromic activation pressure data. Bars represent the average value (also used, e.g., for the bubble chart in **Figure 2c**) over at least three zones; upper and lower extremes are also rendered through (asymmetric) whiskers (largest and smallest values reported by the test campaign, for ease of consultation refer to **Figure SI 11**). Several SP-doping concentrations were considered (see legend); PDMS (2.5:1, 2% wt. SP-doping) provided the lowest activation threshold (around 1.17 ± 0.25 MPa). The polysiloxane prepolymer/curing agent weight ratio composition on the x-label stands for PDMS, while Ds20 is Dragonskin20.

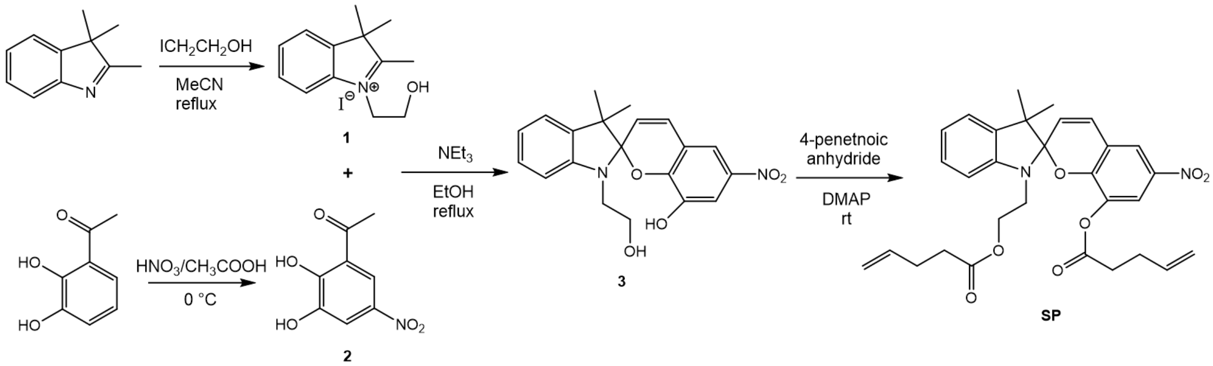

**Figure SI 5** Reaction scheme for the synthesis of **SP**.

**Synthesis of 1)** 2-iodoethanol (7.4 mL, 1.5 equiv.) was added dropwise over 5 min to a solution of 2,3,3-trimethylindolenine (10.0 g, 63 mmol, 1 equiv.) dissolved in 100 mL of dry acetonitrile. The reaction mixture was in a flask, connected to an allihn condenser, and heated to reflux (70°C) and stirred overnight in air atmosphere. Obtained mixture was cooled in an ice bath, added to *n*-hexane (50 mL) in a separation funnel and gently shaked. The product was recovered in the bottom purple phase after ~20 min and dried by rotavapor, while the residual 2-iodoethanol was eliminated in the top yellow phase. The product 2-hydroxyethyl-2,3,3-trimethyl-3H-indolium iodide (**1**) was obtained as a light pink solid. The obtained product was further purified by phase separation in *n*-hexane/acetonitrile twice. Final yield after purification was 17.3 g (yield 83%).

**Synthesis of 2)** 2,3-dihydroxybenzaldehyde (8.0 g, 53 mmol) was dissolved in 50 mL acetic acid and 3 mL of DI water. The solution was cooled to 0°C, then a solution of nitric acid/acetic acid (5 mL/15 mL) was carefully added dropwise over 50 min in a round bottom flask. After stirring for 1.5 hr, the solution was diluted with water, filtered, the precipitate washed with water and dried. The final product 2,3-dihydroxy-5-nitrobenzaldehyde (**2**) was a yellow solid (6.9 g, yield 66%).

**Synthesis of 3)** 15.0 g of **1** (45 mmol) and 8.4 g of **2** (43 mmol) were dissolved in a round bottom flask in absolute ethanol (150 mL). Triethylamine ($NEt_3$, 13.1 ml) was added dropwise over 5 min under stirring until the mixture turns black. The flask was then connected to an allihn condenser, heated to 76°C and stirred overnight. Then the reaction mixture was cooled in an ice bath and purified over a silica column (liquid phase *n*-hexane/ethyl acetate 5:1 v/v) to obtain the product 1'-(2-hydroxyethyl)-3',3'-dimethyl-6-nitrospiro[chromene-2,2'-indolin]-8-ol (**3**) (4.0 g, yield 25%).

**Synthesis of SP)** A mixture of **3** (2.0 g, 5 mmol) and 4-dimethylaminopyridine (DMAP) (0.3 g) was dissolved in 56 mL of dry dichloromethane and stirred for 5 min. 4-pentenoic anhydride (2.6 mL) was added dropwise at room temperature in 3 separate aliquots, waiting for 15 min after each addition in an ice bath. The solution changed from blue to purple and it was stirred overnight at room temperature. Then, the reaction mixture was poured into a separation funnel and extracted with a saturated sodium bicarbonate solution (75 mL). The organic layer was extracted with 1 N HCl solution (75 mL), which turned yellow, and washed with water. The organic layer was then extracted with brine, and further dried over solid sodium sulfate. The dark purple solution was concentrated by rotavapor to give a dark purple oil. Then boiled Pet-ether (500 mL) was poured into the purple oil, resulting in a yellowish solution. Finally, the product was further purified over a neutral $Al_2O_3$ column (liquid phase dicloromethane) and then rotavaped giving the reddish oily product 3',3'-dimethyl-6-nitro-1'-(2-(pent-4-enoyloxy)ethyl)spiro[chromene-2,2'-indolin]-8-yl pent-4-enoate (**SP**) (1.5 g, yield 55%).

At the polymeric level, the SP-beared with vinyl groups cross-links into the elastomeric networks by platinum catalyzed hydrosilylation reaction. Indeed, thanks to the bis-alkene functionalization groups (**SP**), this reaction reveals as the same curing reaction as that between vinyl- and hydrosilane functionalities in the SP-free silicone networks.

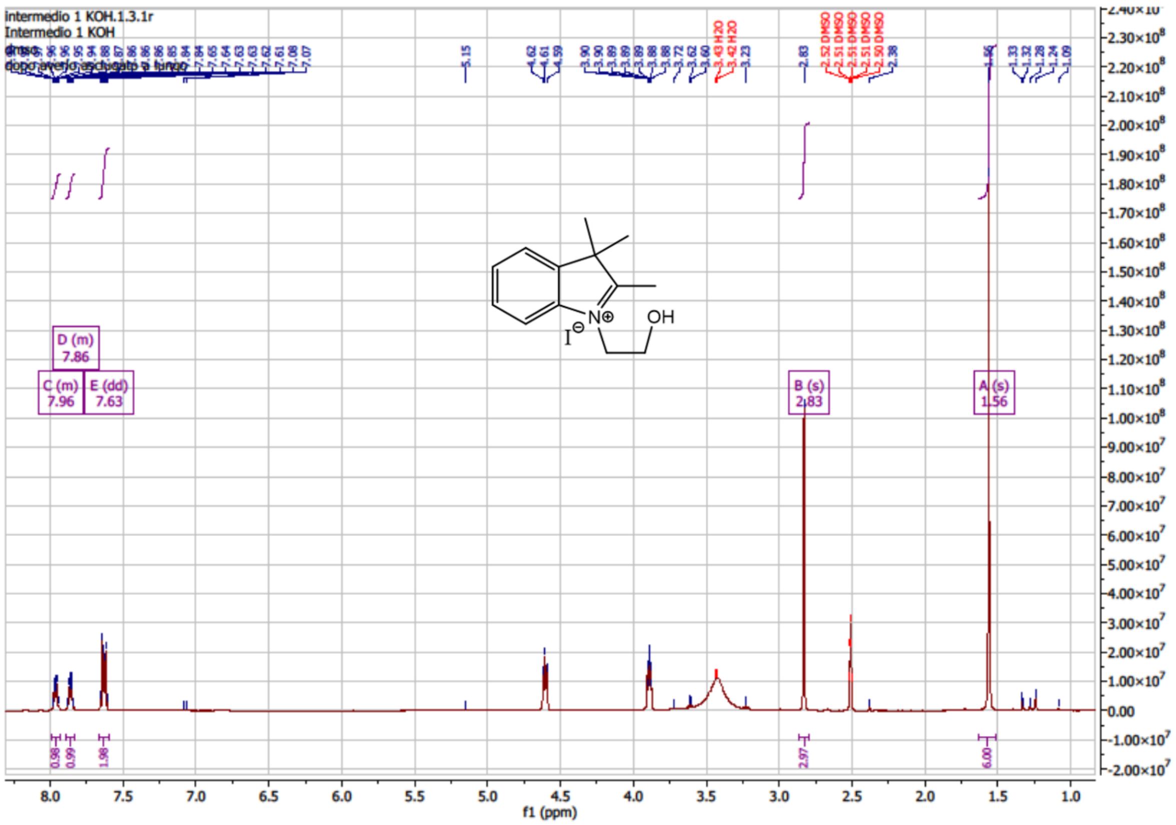


**Figure SI 6** $^{1}$H NMR (400 MHz, DMSO-d6) δ 7.97-7.94 (m, 1H), 7.88-7.84 (m, 1H), 7.63 (dd, 2H), 4.62-4.59 (m, 2H), 3.90-3.88 (m, 2H), 2.83 (s, 3H), 1.56 (s, 6H).

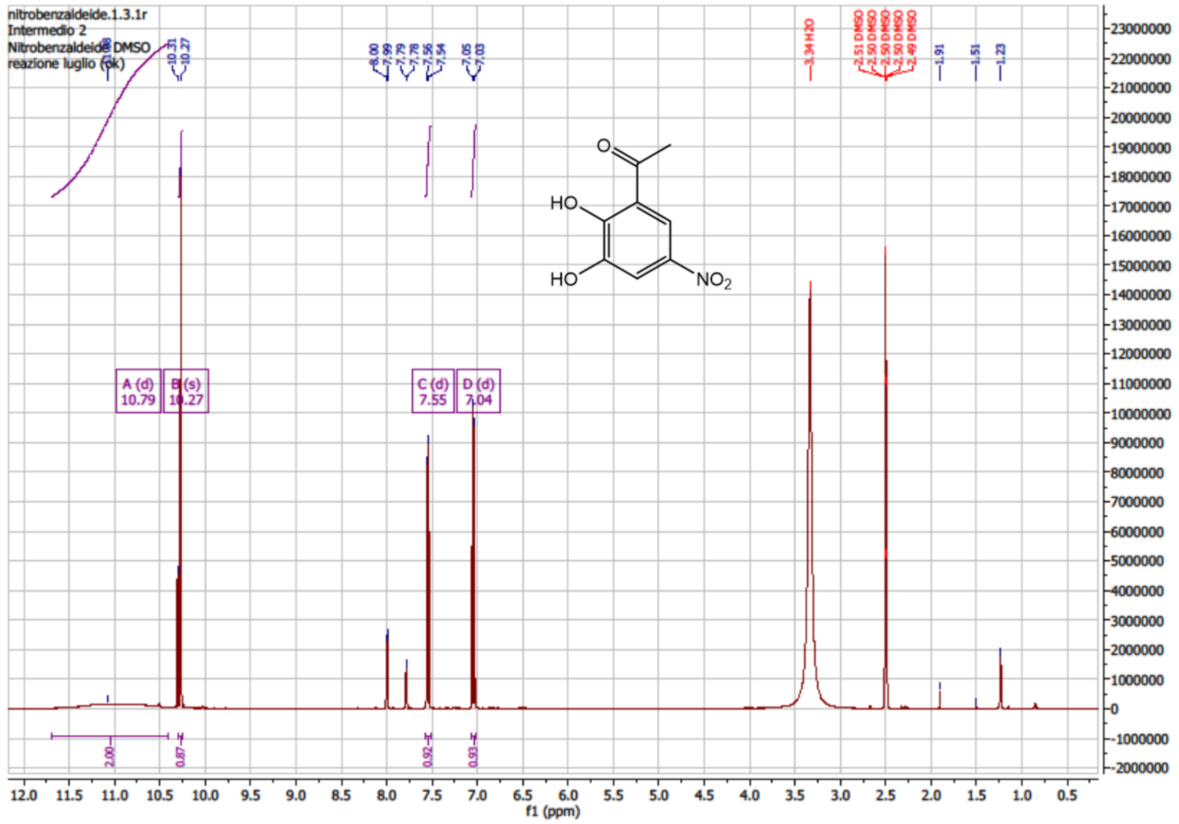


**Figure SI 7** $^{1}$H NMR (400 MHz, DMSO-d6) δ 10.79 (d, 2H), 10.27 (s, 1H), 7.55 (d, 1H), 7.04 (d, 1H).

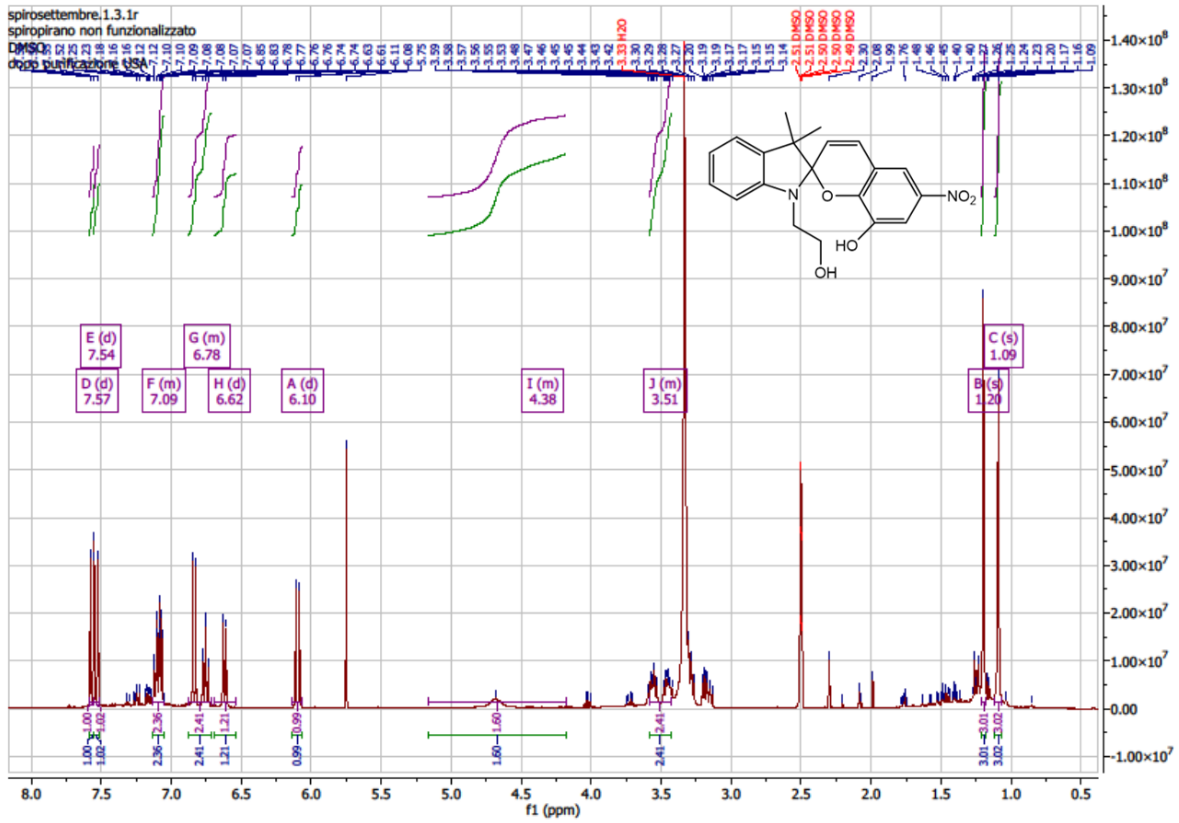

**Figure SI 8** $^{1}$H NMR (400 MHz, DMSO-d6) δ 7.57 (d, 1H), 7.54 (d, 1H), 7.25-7.07 (m, 2H), 6.85-6.74 (m, 2H), 6.62 (d, 1H), 6.10 (d, 1H), 4.6 (b, 2H), 3.59-3.42 (m, 2H), 1.20 (s, 3H), 1.09 (s, 3H).

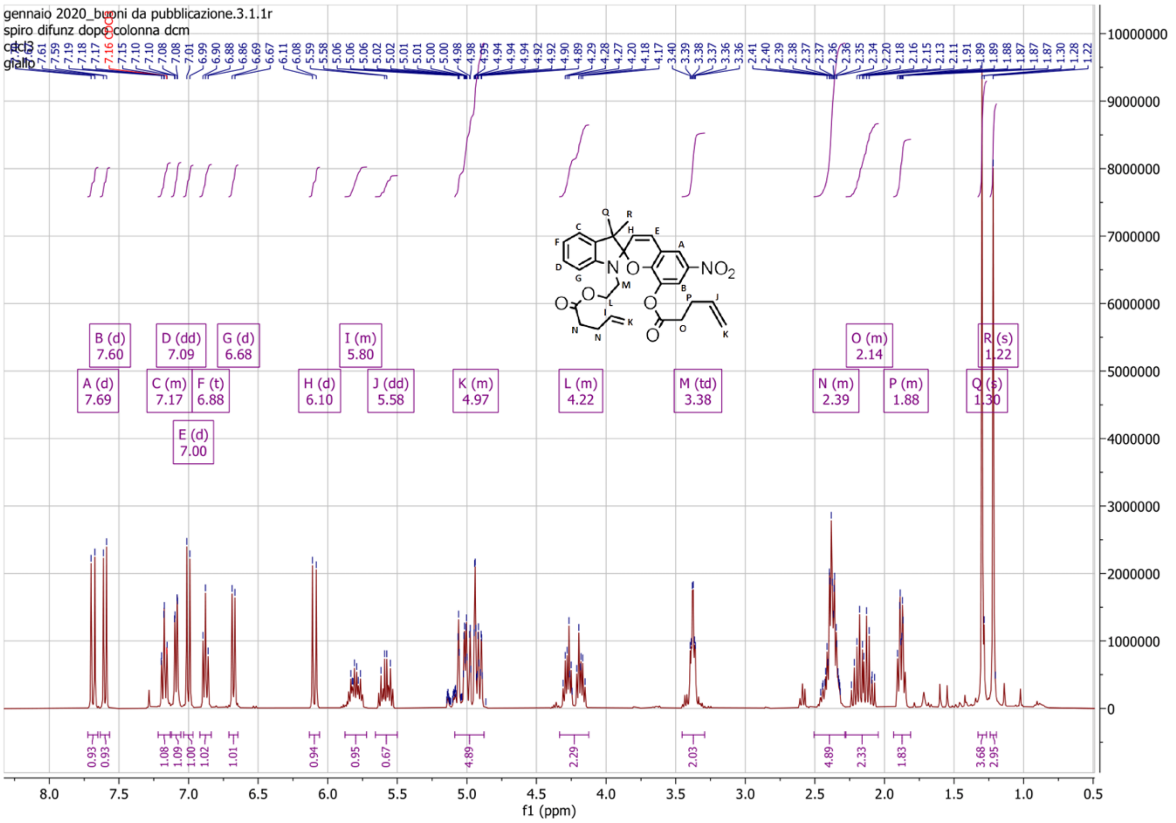
gennaio 2020_buoni da pubblicazione.3.1.1r
spiro difunz dopo colonna dcm
cdcl3
giallo
7.61 7.59 7.19 7.18 7.17 7.16 CDCl3 7.15 7.10 7.08 7.08 7.01 6.99 6.90 6.88 6.86 6.69 6.67 6.11 6.08 5.59 5.58 5.06 5.06 5.06 5.02 5.02 5.01 5.01 5.00 5.00 4.98 4.98 4.95 4.94 4.94 4.94 4.92 4.92 4.90 4.89 4.29 4.28 4.27 4.20 4.18 4.17 3.40 3.39 3.38 3.37 3.36 2.41 2.40 2.39 2.38 2.37 2.37 2.36 2.36 2.35 2.34 2.20 2.18 2.16 2.15 2.13 2.11 1.91 1.89 1.89 1.88 1.87 1.87 1.30 1.28 1.22
NO2
A (d) 7.69
B (d) 7.60
C (m) 7.17
D (dd) 7.09
E (d) 7.00
F (t) 6.88
G (d) 6.68
H (d) 6.10
I (m) 5.80
J (dd) 5.58
K (m) 4.97
L (m) 4.22
M (td) 3.38
N (m) 2.39
O (m) 2.14
P (m) 1.88
Q (s) 1.30
R (s) 1.22
0.93 0.93 1.08 1.09 1.00 1.02 1.01 0.94 0.95 0.67 4.89 2.29 2.03 4.89 2.33 1.83 3.68 2.95
f1 (ppm)

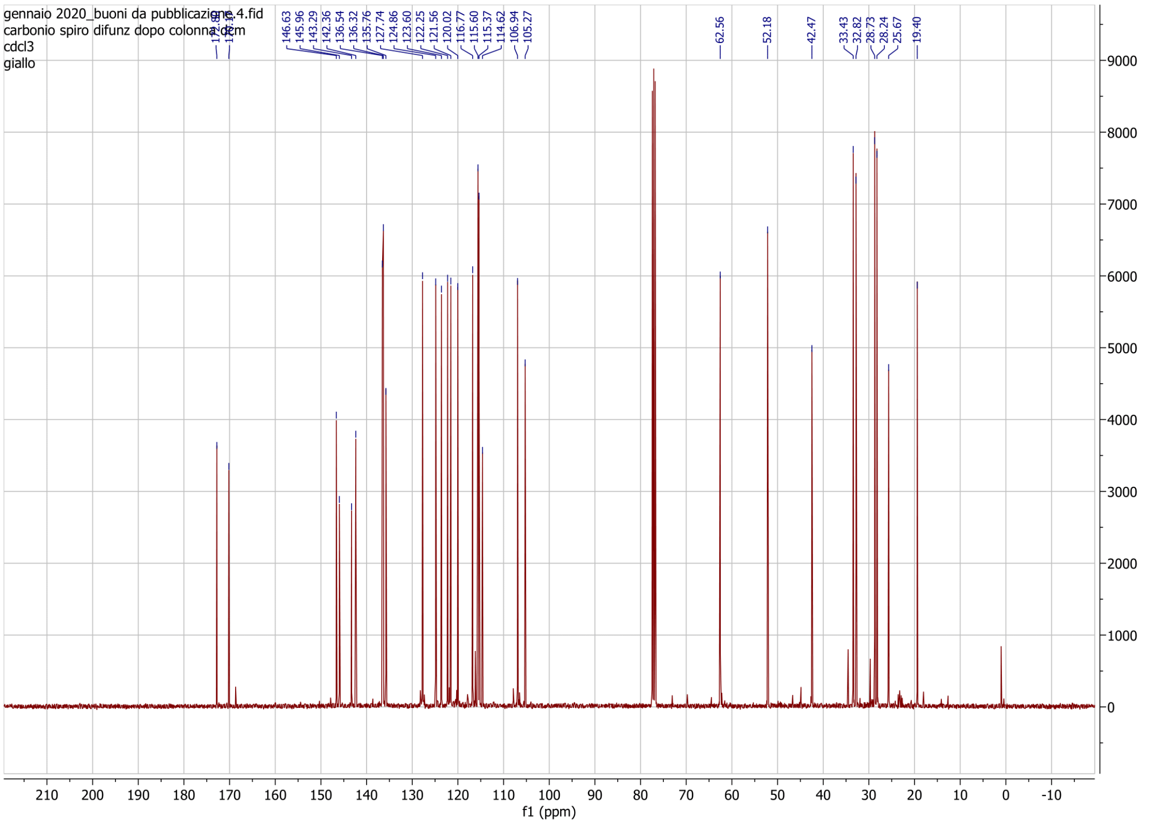
gennaio 2020_buoni da pubblicazione.4.fid
carbonio spiro difunz dopo colonna dcm
cdcl3
giallo
146.63 145.96 143.29 142.36 136.54 136.32 135.76 127.74 124.86 123.60 122.25 121.56 120.02 116.77 115.60 115.37 114.62 106.94 105.27 62.56 52.18 42.47 33.43 32.82 28.73 28.24 25.67 19.40
f1 (ppm)

**Figure SI 9** $^{1}H$ NMR (400 MHz, $CDCl_3$) δ 7.69 (d, 1H), 7.60 (d, 1H), 7.19-7.10 (m, 1H), 7.09 (dd, 1H), 7.00 (d, 1H), 6.88 (t, 1H), 6.68 (d, 1H), 6.10 (d, 1H), 5.85-5.75 (m, 1H), 5.58 (dd, 1H), 5.06-4.89 (m, 4H), 4.29-4.17 (m, 2H), 3.38 (td, 2H), 2.41-2.34 (m, 4H), 2.20-2.11 (m, 2H), 1.91-1.87 (m, 2H), 1.30 (s, 3H), 1.22 (s, 3H).

$^{13}C$ NMR (400 MHz, $CDCl_3$) δ 172.8, 170.17, 146.63, 145.96, 143.29, 142.36, 136.54, 136.32, 135.76, 127.74, 124.86, 123.60, 122.25, 121.56, 120.02, 116.77, 115.60, 115.37, 114.62, 106.94, 105.27, 62.56, 52.18, 42.47, 33.43, 32.82, 28.73, 28.24, 25.67, 19.40.

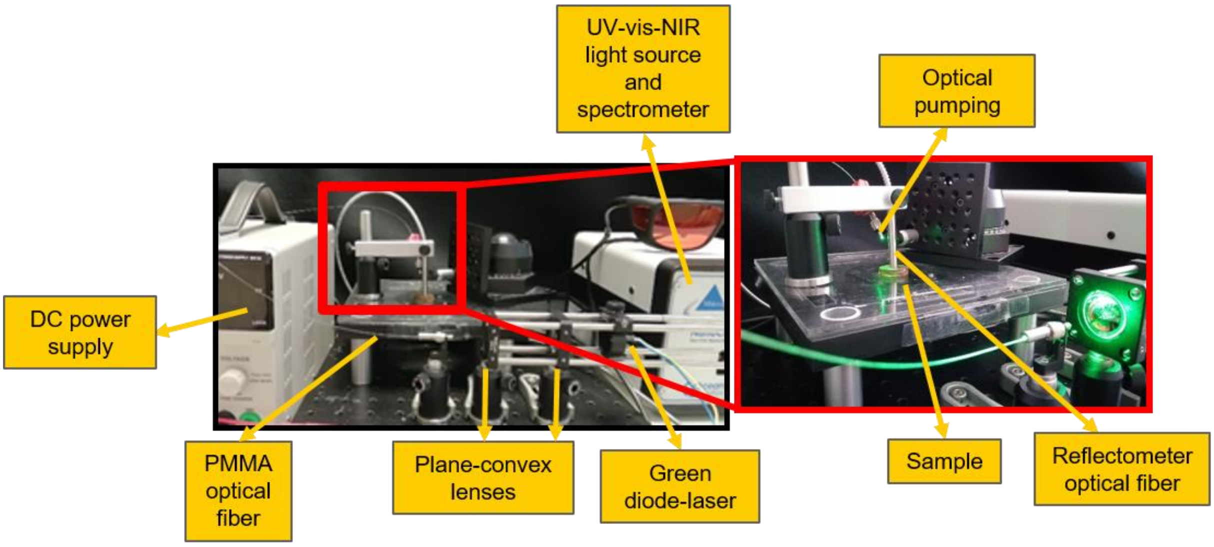


**Figure SI 10** A picture of the optical setup outside the black box, and under ambient light. The laser was activated for ease of visualization.

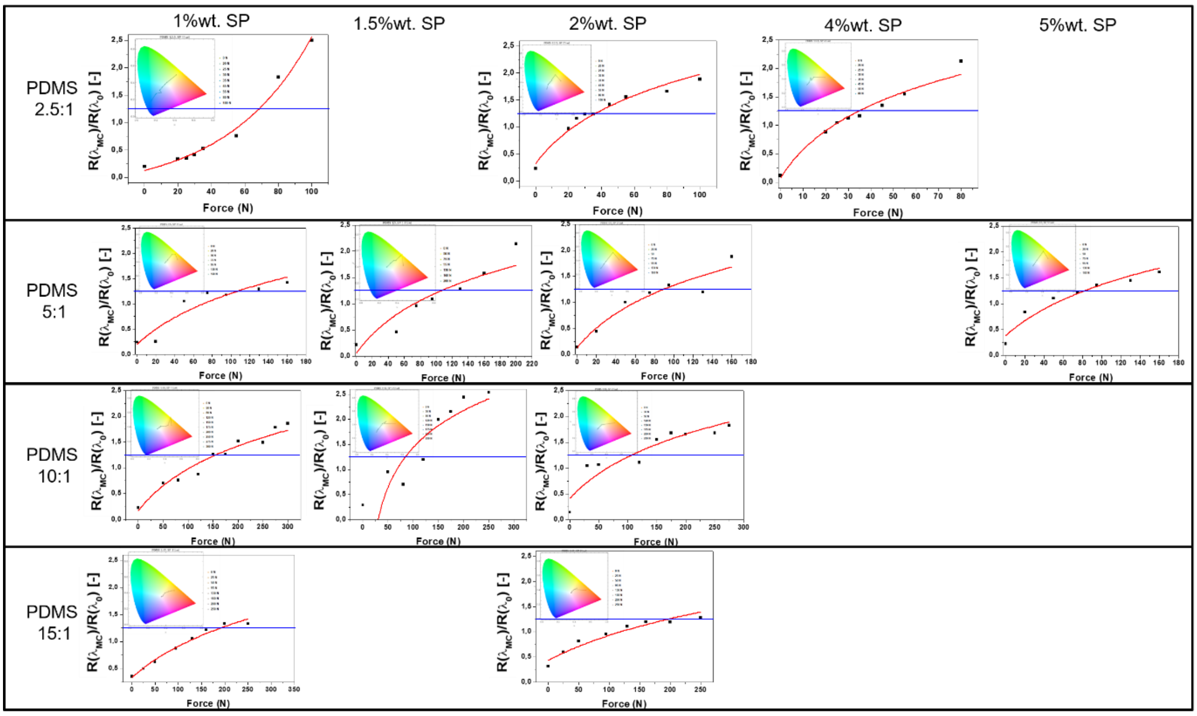


**Figure SI 11** Ratio $R(\lambda_{MC})/R(\lambda_0)$, at varying indentation forces, for each PDMS prepolymer/curing agent ratio vs. %wt. SP-doping (cured at 90 °C for 9 h). All the averaged data (minimum n=3) per each force were reported (squared dots). A logarithmic trend fitted well the experimental data (red line). The minimum averaged mechanochromic activation force (reported in **Figure 2c** and **Figure SI 4**) was the mean value between three points, i.e. when $R(\lambda_{MC})/R(\lambda_0)$={1.15, 1.25, 1.50} (for ease of visualization $R(\lambda_{MC})/R(\lambda_0)$=1.25 was highlighted with a blue line). The asymmetric whiskers in **Figure SI 4** correspond to the averaged force/pressure at $R(\lambda_{MC})/R(\lambda_0)$=1.15 (lower limit) and $R(\lambda_{MC})/R(\lambda_0)$=1.50 (upper limit) in the plot. Data refer to the CIE-1931 colorimetric diagram.

***Mechanochromic activation pressure at variable temperatures:***

From the experimental results we can speculate that, for $k_BT<E_{a1}<E_{a2}^*$ or $E_{a1}<k_BT<E_{a2}^*$, the mechanical energy is still the principal mechanism that provides energy equal than SP->MC (T=25°C or T=60°C). While if $E_{a1}< E_{a2}^*< k_BT$, the occupation of the states in the potential wells is regulated most likely from the Boltzmann statistics, decreasing the ratio of the bluish band collected by the spectrophotometer (T=100°C). For T<60°C, the developed solution shows no thermal dependency as regard the minimum mechanochromic activation pressure. Therefore, the human body temperature should not induce drifts affecting sensor behavior.

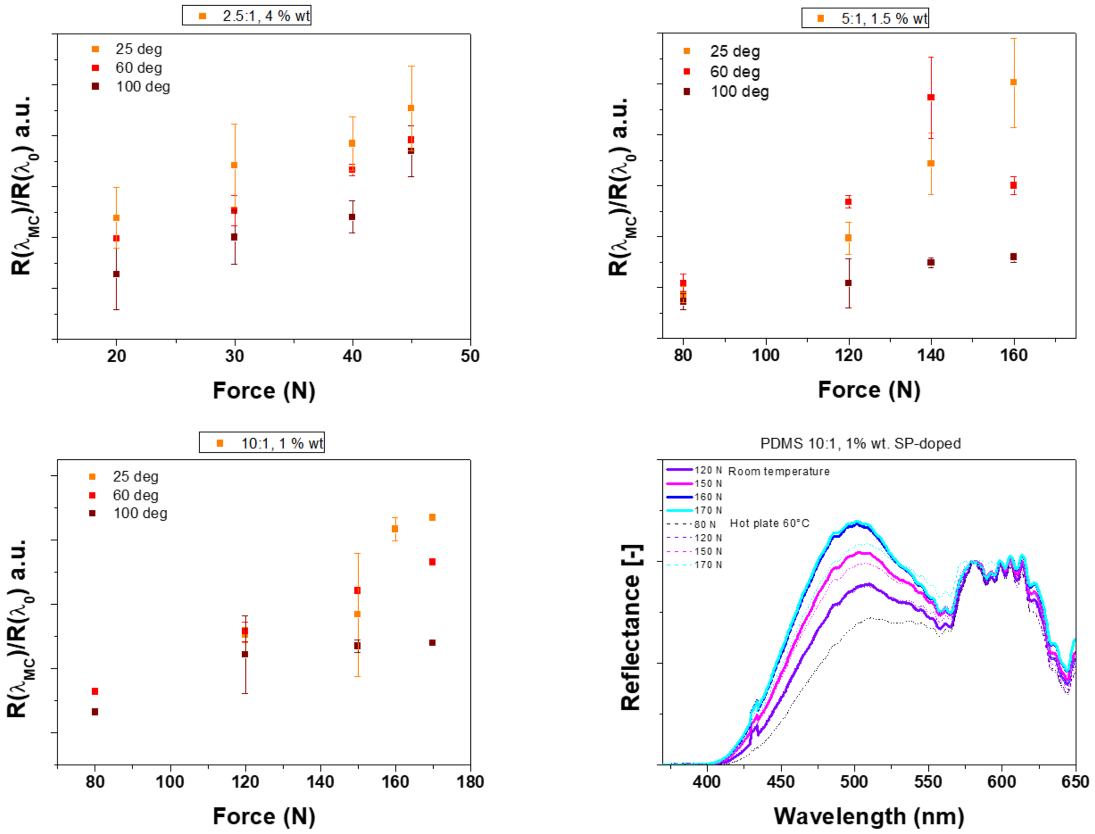


**Figure SI 12** Mechanochromic activation pressure at variable temperature for PDMS samples 2.5:1, 4%wt. SP-doped, 5:1, 1.5% wt. SP-doped, and 10:1, 1% wt. SP-doped, and reflectance spectra at room temperature and at 60°C for PDMS 10:1, 1% wt. SP-doped. We assumed the indentation force values equal to the mechanochromic pressure activation calculated at room temperature (**Figure 2c**, **Figure SI 4**, **Figure SI 11**). For each sample, n=2 test repetitions were evaluated, and data are shown with the error bars (mean ± SD). The mechanochromic activation pressure dependency at T=25°C and T=60°C is almost similar within the error bars. The decrease of the absolute value of the $R(\lambda_{MC})/R(\lambda_0)$ at the higher temperature (T=100°C) was given by the longer spectrophotometer integration time than the temperature-dependent recovery time (i.e. reduced by high thermal treatment). Spectral data (normalized at the dominant wavelength) are reported for PDMS 1:10, 1% wt. SP-doped at room temperature, as well as for thermal annealing at 60°C.

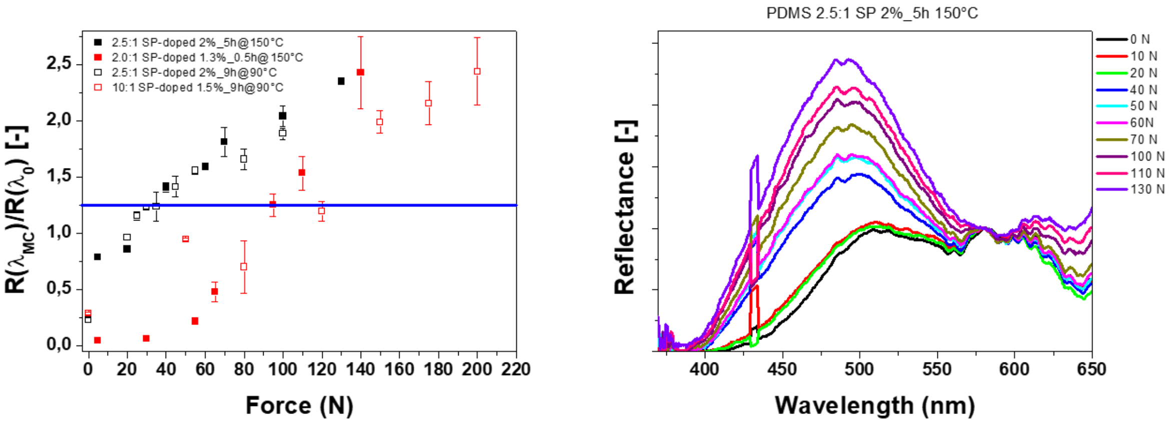


**Figure SI 13** On the left: mechanochromic activation force threshold for PDMS 2.0:1,1.3% wt. SP-doped cured at 150°C for 0.5 h, and PDMS 2.5:1, 2% wt. SP-doped cured at 150°C for 5 h (black and red filled squared dots, n=3, mean ± SD), considering the $R(\lambda_{MC})/R(\lambda_0)$. Black and red empty squared dots represent averaged data (n=3, mean ± SD) for PDMS 2.5:1, 2% wt. SP-doped cured at 90°C for 9 h, and PDMS 10:1, 1.5% wt. SP-doped cured at 90°C for 9 h, with comparable elastic modulus, and % SP loading of the previous compositions (please refer to **Table 1**). The averaged mechanochromic activation force was comparable in samples with similar elastic modulus and mechanophore loading. This result corroborates the positive correlation between the mechanical properties of the host matrix and the mechanophore incorporation in the network (at the given boundary condition, i.e. curing temperature, curing time, prepolymer/curing agent, mechanophore concentration). On the right: reflectance spectra by varying indentation force for PDMS 2.5:1, 2% wt. SP-doped cured at 150°C for 5 h.

**Table SI 1**. Descriptive statistics analysis for n=5 test repetition for each temperature sample.

| Material Type, Prepolymer/curing agent weight ratios, %wt. SP | Temperature (°C) | Mean (s) | Standard deviation (s) | Median (s) | Range (s) |
|---|---|---|---|---|---|
| PDMS, 2.5:1, 2% | 20 | 207 | 28.3 | 209 | 68 |
| PDMS, 2.5:1, 2% | 40 | 81.6 | 17 | 82 | 44 |

| | | | | | |
|---|---|---|---|---|---|
| PDMS, 2.5:1, 2% | 60 | 41.4 | 16.5 | 39 | 45 |
| PDMS, 2.5:1, 2% | 80 | 19.8 | 16.4 | 16 | 41 |
| PDMS, 2.5:1, 2% | 100 | 7.4 | 7.7 | 4.9 | 18.7 |

**Table SI 2**. Chromogenic recovery time measurement at room temperature, with the fitted parameter on the experimental data acquired, by means of OriginPro 8.1 (OriginLab®).

| Material Type, Prepolymer/curing agent weight ratios, % wt. SP doping | Fit equation (y = A1*exp(-x/t1) + y0) | y0 | A1 | t1 | Adj. R-square |
|---|---|---|---|---|---|
| PDMS, 2.5:1, 1% | - | 0.24 | 0.76 | 207.99 | 0.997 |
| PDMS, 2.5:1, 2% | - | 0.05 | 0.95 | 27.55 | 0.996 |
| PDMS, 2.5:1, 4% | - | 0.13 | 0.87 | 313.05 | 0.939 |
| PDMS, 5:1, 1% | - | -0.11 | 1.10 | 1146.46 | 0.993 |
| PDMS, 5:1, 1.5% | - | 0.59 | 0.42 | 53.38 | 0.979 |
| PDMS, 5:1, 5% | - | 0.46 | 0.53 | 1486.05 | 0.956 |
| PDMS, 10:1, 1% | - | -0.01 | 1.01 | 116.43 | 0.994 |

| | | | | | |
|---|---|---|---|---|---|
| | | 0.47 | 0.52 | 271.26 | 0.968 |
| PDMS, 10:1, 1.5% | - | | | | |
| | | 0.41 | 0.58 | 31.38 | 0.996 |
| PDMS, 10:1, 2% | - | | | | |
| | | 0.11 | 0.87 | 236.31 | 0.923 |
| PDMS, 15:1, 1% | - | | | | |
| | | -7.39E-04 | 0.98 | 559.55 | 0.985 |
| PDMS, 15:1, 2% | - | | | | |

**Representative pressures/force for surgical tissue grasping/retraction**

Stress thresholds for tissue damage were retrieved from literature (where porcine data are generally deemed functional for subsequent in-vivo tests), as relevant for tissue grasping. As shown in **Table 2**, the considered threshold is around 140-240 kPa. Furthermore, we collected relevant retraction force data related to human organs. **Table 3** summarizes the aforementioned data, as complemented with corresponding grasping forces obtained by assuming a friction coefficient μ (two values of such a coefficient were considered, for illustration). Human organs manipulation required slight forces ranging from a minimum of about 0.5 N for pancreas to about 30 N for uterus. Robotic systems need to adopt a gentle touch when firmly grasping human organs: the proposed mechanochromic grasper can support the achievement of the sought safe and effective clutching.
